# The dynamics of three-dimensional chromatin organization and phase separation in cell fate transitions and diseases

**DOI:** 10.1186/s13619-022-00145-4

**Published:** 2022-12-21

**Authors:** Xiaoru Ling, Xinyi Liu, Shaoshuai Jiang, Lili Fan, Junjun Ding

**Affiliations:** 1 Advanced Medical Technology Center, The First Affiliated Hospital, Zhongshan School of Medicine, Sun Yat-Sen University, Guangzhou, Guangdong China; 2 RNA Biomedical Institute, Sun Yat-Sen Memorial Hospital, Zhongshan School of Medicine, Sun Yat-Sen University, Guangzhou, Guangdong China; 3 Center for Stem Cell Biology and Tissue Engineering, Key Laboratory for Stem Cells and Tissue Engineering, Ministry of Education, Zhongshan School of Medicine, Sun Yat-Sen University, Guangzhou, Guangdong China; 4 Guangzhou Key Laboratory of Formula-Pattern of Traditional Chinese Medicine, School of Traditional Chinese Medicine, Jinan University, Guangzhou, Guangdong China; 5 Department of Histology and Embryology, School of Basic Medical Sciences, Guangzhou Medical University, Guangzhou, 511436 China; 6 West China Biomedical Big Data Center, West China Hospital, Sichuan University, Chengdu, 610041 China

**Keywords:** 3D chromatin organization, Phase separation, Cell fate transitions, Disease

## Abstract

Cell fate transition is a fascinating process involving complex dynamics of three-dimensional (3D) chromatin organization and phase separation, which play an essential role in cell fate decision by regulating gene expression. Phase separation is increasingly being considered a driving force of chromatin folding. In this review, we have summarized the dynamic features of 3D chromatin and phase separation during physiological and pathological cell fate transitions and systematically analyzed recent evidence of phase separation facilitating the chromatin structure. In addition, we discuss current advances in understanding how phase separation contributes to physical and functional enhancer-promoter contacts. We highlight the functional roles of 3D chromatin organization and phase separation in cell fate transitions, and more explorations are required to study the regulatory relationship between 3D chromatin organization and phase separation.

**Graphical Abstract:**

3D chromatin organization (shown by Hi-C contact map) and phase separation are highly dynamic and play functional roles during early embryonic development, cell differentiation, somatic reprogramming, cell transdifferentiation and pathogenetic process. Phase separation can regulate 3D chromatin organization directly, but whether 3D chromatin organization regulates phase separation remains unclear.
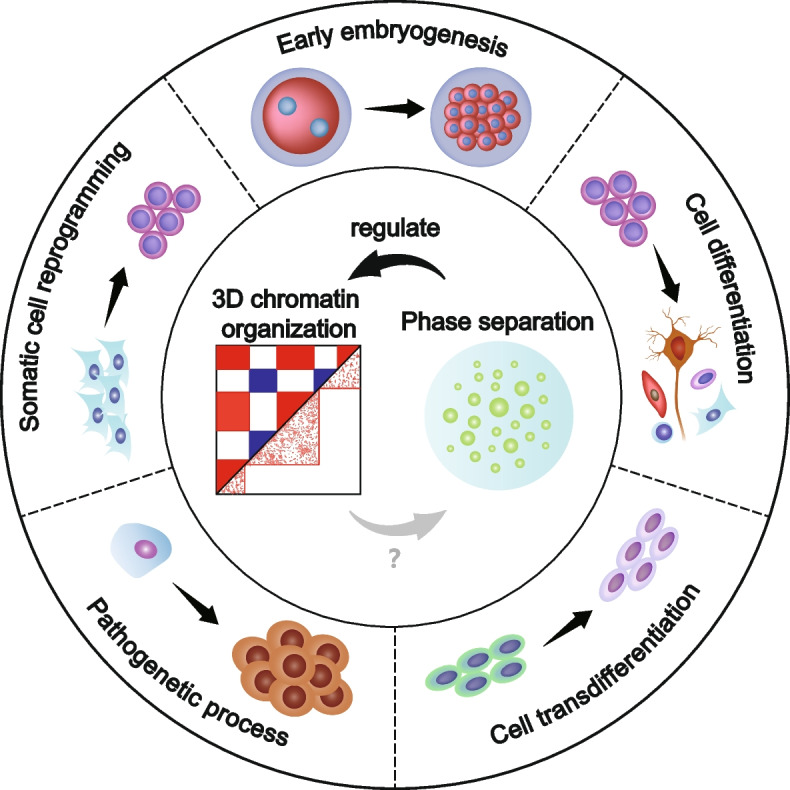

## Background

Cell fate transitions are a set of important biological processes in multicellular organisms that determine the functions of different cell types. Numerous molecular mechanisms are associated with cell fate regulation, such as epigenetic landscape remodeling, 3D chromatin reorganization, and phase separation (Atlasi and Stunnenberg 2017; Grosch et al. 2020; So et al. 2021; Vallot and Tachibana 2020; Zhao et al. 2021; Zheng and Xie 2019). Epigenetic modification is a major regulator of early embryogenesis and developmental process by mediating gene transcription (Morgan and Shilatifard 2020; Reik 2007). Likewise, 3D chromatin architecture and phase separation dynamics have emerged as new mechanisms in cell fate regulation (Boija et al. 2021; Boltsis et al. 2021; So et al. 2021). The molecular basis of cell fate control is also a clinically significant issue to be answered and provides potential therapeutic targets. In leukemogenesis, abnormal phase separation of known tumorigenic chimeras induces the misfolding of chromatin loop and potentiates oncogene activation (Ahn et al. 2021). The development of novel therapeutic approaches to regulate phase transitions may be instrumental in treating such diseases associated with aberrant condensates. However, the mechanisms underlying the contribution of 3D chromatin organization and phase separation to cell fate transition remain unclear.

The 3D chromatin architecture undergoes considerable alterations in accordance with changes in gene expression during cell fate transitions (Vallot and Tachibana 2020; Zhao et al. 2021; Zheng and Xie 2019). Chromatin conformation capture (3C)-based techniques (e.g., Hi-C), which work by proximity ligation, have been critical for the rapid development in the study of genome-wide 3D chromatin structure (Dekker et al. 2002; Dostie et al. 2006; Lieberman-Aiden et al. 2009; Simonis et al. 2006; Zhao et al. 2006). There are a few other powerful techniques for capturing chromatin interactions (Jerkovic and Cavalli 2021; Kempfer and Pombo 2020), such as split-pool recognition of interactions by tag extension (SPRITE) (Quinodoz et al. 2018), genome architecture mapping (GAM) (Beagrie et al. 2017), and imaging approaches (Bintu et al. 2018; Maslova and Krasikova 2021; Su et al. 2020; Wang et al. 2016b). The 3D organization of mammalian genome can be divided into the following structures: chromosome territories, transcriptionally active A and transcriptionally inactive B compartments, topologically associating domains (TADs), and chromatin loops (Dixon et al. 2012; Nora et al. 2012; Rao et al. 2014; Zheng and Xie 2019). TADs refer to chromatin domains with a higher frequency of intra-domain interactions than that of inter-domain interactions, including cohesin-dependent and cohesin-independent domains. Based on the hierarchy of inter-TAD contacts, TADs can be further merged into high-order domains called metaTADs (Fraser et al. 2015). Different cell types exhibit heterogeneous chromatin folding maps that determine gene expression patterns specific to each cell type (Schmitt et al. 2016). Compartment shifting, metaTAD, and TAD reorganization, and chromatin loop dynamics are common 3D changes, which display distinct features and correlate with transcriptional changes during biological and pathological processes (Chen et al. 2019; Dixon et al. 2015; Fraser et al. 2015; Hnisz et al. 2016; Northcott et al. 2014; Zhao et al. 2021). 3D chromatin reorganization is now recognized as a critical contributor to cell fate decision, although most of the regulatory mechanisms remain to be explored.

Dynamics of phase separation in both nucleus and cytoplasm is another interesting and widespread phenomenon during cell fate transitions (Grosch et al. 2020; So et al. 2021). Intracellular membrane-less organelles or biomolecular condensates, such as germ granules, stress granules, and nuclear bodies (Banani et al. 2017; Protter and Parker 2016; Sabari et al. 2020), are mainly composed of aggregated proteins and RNAs via phase separation. They have diverse and crucial functions, including but not limited to mRNA regulation, chromatin organization, and gene expression regulation. In the nucleus, liquid-liquid phase separation of transcription coactivator BRD4 and Mediator can drive the formation of transcriptional condensates at super-enhancers, large clusters of enhancers, to facilitate gene activation (Boija et al. 2018; Cho et al. 2018; Sabari et al. 2018). Biomolecular condensates are highly dynamic, and undergoes assembly, disassembly, fusion, isolation, and changes in components, condensation and subcellular localization during different cell fate transitions (Banani et al. 2017; Boija et al. 2021; Liu et al. 2020a; Sabari et al. 2020; So et al. 2021). Numerous evidences have highlighted the possible functional and multifaceted role of phase separation events in basic biological processes, especially in early embryogenesis, germ cell development and diseases (Quiroz et al. 2020; Spannl et al. 2019).

Recent studies show that phase separation can regulate 3D chromatin assembly (Liu et al. 2021; Shin et al. 2019; Wang et al. 2021; Wei et al. 2022). On one hand, two models for phase-separated chromatin compartmentalization based on different mechanisms have been proposed (Erdel and Rippe 2018). One is liquid-liquid phase separation based on the weak multivalent interactions of chromatin binding factors. The other model is polymer-polymer phase separation (PPPS) stabilized by DNA-bridging proteins that cross-link different chromatin segments, such as DNA-cohesin clustering through the DNA-cohesin-DNA bridges (Ryu et al. 2021). On the other hand, the disruption of distinct condensates leads to aberrant chromatin folding in some diseases (Ahn et al. 2021; Shi et al. 2021). Phase separation ability of the pluripotent factor OCT4 contributes to somatic cell reprogramming by regulating TAD reorganization (Wang et al. 2021). Coincidentally, through induced-phase separation, the structural factor CTCF can mediate inter-A compartment interactions, promote self-renewal of ESCs and suppress neural differentiation (Wei et al. 2022). However, whether nuclear condensates affect cell fate transitions by manipulating 3D chromatin reorganization is a widespread mechanism remains misty. To this end, we have systematically reviewed the dynamics of 3D chromatin organization and phase separation during cell fate transitions and the pathogenesis of various diseases. The relationship between 3D chromatin organization and nuclear phase separation has also been discussed, along with new strategies emerging in recent studies.

## Dynamics of 3D chromatin organization and phase separation during cell fate transitions and diseases

### Early embryonic development

#### Chromatin organization

**Fig. 1:**
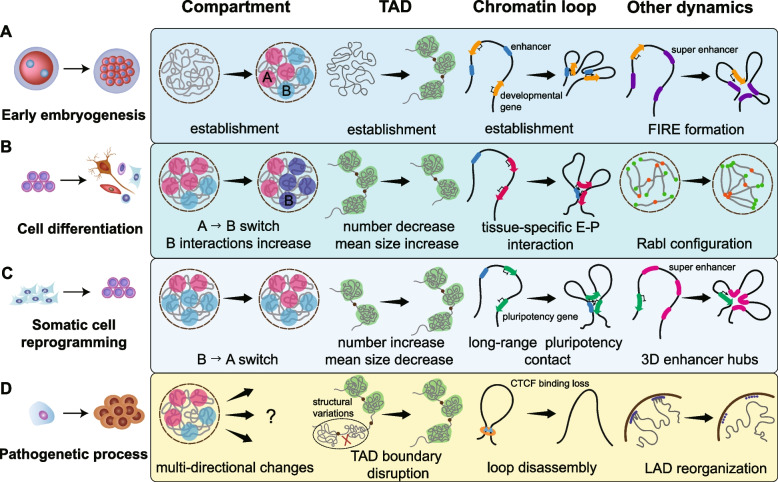
Dynamics of 3D chromatin organization during different cell fate transitions and diseases. **A** During early embryogenesis, compartments, TADs and chromatin loops are reestablished at species-specific stage; frequently interacting regions (FIREs) are identified as well, within which super-enhancers are enriched. **B** During cell differentiation, A-to-B compartment shift and interactions within B compartments increase; TAD number decreases and TAD size increases; tissue-specific E-P interactions are established consistent with cell-specific gene expression; increased centromere and telomere clusters (Rabl configuration) are formed. **C** During somatic cell reprogramming, the proportion of A compartments are slightly increased; TAD dynamics are opposed to that in cell differentiation; long-range pluripotency contacts and 3D enhancer hubs are formed. **D** During pathogenetic process, compartments undergo muti-directional and complex dynamics in different diseases; TAD boundary disruption are mainly caused by structural variations at boundary loci; loss of CTCF binding lead to loop disassembly; Lamin-associated domains are reorganized, such as abnormal reduction in HGPS fibroblast cells

The 3D chromatin organization is dramatically reconstructed (Fig. 1A) during early mammalian and non-mammalian embryonic development (Chen et al. 2019; Du et al. 2017; Flyamer et al. 2017; Hug et al. 2017; Kaaij et al. 2018; Ke et al. 2017; Nakamura et al. 2021; Niu et al. 2021; Sun et al. 2021; Wike et al. 2021). After fertilization, the A/B compartments, TADs, and chromatin loops are largely absent for species-specific duration, which corresponds to a transcriptionally-inactive state. Zygotic genome activation (ZGA) is a crucial event denoting the initiation of gene expression (Jukam et al. 2017) and is accompanied by 3D chromatin reestablishment (Hug and Vaquerizas 2018; Li et al. 2019). The exact time point of this restructuring depends on the developmental rates of the species.

High-order chromatin structure is gradually established during early embryogenesis in human (Chen et al. 2019). In human 2-cell embryos, the genome is under an unstructured state; compartments and TADs start emerging until the 8-cell stage and become increasingly evident at the blastocyst stage. TAD boundaries are mainly established after the beginning of ZGA with the sharp expression of CCCTC-Binding Factor (CTCF). As an important structural factor, CTCF contributes to the establishment of TADs and partially maintains cell type-specific frequently interacting regions (FIREs) in A compartments (Fig. 1A). FIREs are mainly composed of super-enhancers which are clusters of enhancers in close genomic proximity with high levels of transcription factors or Mediator binding (Whyte et al. 2013). The TAD boundaries become more fixable during developmental progress. In the mouse embryos, however, compartments and TADs become apparent from 2-cell embryos to 8-cell embryos (Ke et al. 2017). It’s worth noting that two parental genomes exhibit different patterns even at 8-cell stage after convergence (Du et al. 2017).

Early embryogenesis of non-mammalian animals also undergoes de novo assembly of 3D chromatin at ZGA. During *Xenopus tropicalis* embryogenesis, TADs start to emerge at the onset of mid-blastula transition and become consolidated continuously from stage 9 to stage 23 (Niu et al. 2021). This process is followed by progressive compartmentalization and appearance of loops and stripes. In addition to CTCF and Rad21, chromatin remodeling factor ISWI is also required for TAD formation in *X. tropicalis*. In zebrafish or medaka, chromatin structures are absent before ZGA and reestablished during gastrulation (Kaaij et al. 2018; Nakamura et al. 2021; Wike et al. 2021). Consistent with vertebrates, reorganization of 3D chromatin in *Drosophila* also occurs during ZGA (Hug et al. 2017; Sun et al. 2021). Interestingly, TAD formation has been proved to be partly independent of transcription in mice, *X. tropicalis* and *Drosophila*. Therefore, ZGA is an important stage for 3D genome reorganization, but not necessary for all species.

With our understanding in 3D chromatin organization increasing, how it is established during early embryonic development has become an interesting question. Recent studies have identified several contributors of 3D chromatin reconstructing in different species as follows: (1) Different chromatin architecture associating factors are required. The cohesin complex and CTCF are important structural factors regulating TAD establishment through loop extrusion during human embryogenesis (Chen et al. 2019). In the loop extrusion model, cohesin extrudes a DNA loop continuously until it encounters oriented CTCF (Davidson and Peters 2021; Fudenberg et al. 2016). Chromatin remodeling complex ISWI is necessary for de novo TAD formation possibly through mediating CTCF binding in *X. tropicalis* (Niu et al. 2021). Heterochromatin protein 1α (HP1α) and transcription factor Zelda respectively contribute to the formation of B compartments and locus-specific TAD boundaries in *Drosophila* (Hug et al. 2017; Zenk et al. 2021). Although RNA Pol II and transcription factors are enriched at TAD boundaries, transcription inhibition has a limited effect on TAD establishment and decreases TAD insulation markedly in mice and *Drosophila* (Hug et al. 2017; Ke et al. 2017). Therefore, RNA Pol II and transcription factors probably play an essential role in sustaining rather than establishing TADs during early development. Apart from TADs, it is still unclear whether they are directly involved in establishing other 3D chromatin structures in mammals; (2) Transcription is important for development but not stringently required for 3D chromatin establishment. Inhibition of transcription results in 3D chromatin reconstructing failure in human embryos. Distinct from humans, TAD-based chromatin conformation is independent of transcription in mice, *Drosophila,* and *X. tropicalis* (Hug et al. 2017; Ke et al. 2017; Niu et al. 2021). The dependence on transcription is likely influenced by the storage of structural factors in germ cells, which differs across species; (3) In all studied species, 3D chromatin structure is gradually established through several cell divisions, which means cell divisions may play an essential role in 3D chromatin establishment. Inhibition of DNA replication in mouse 2-cell embryos impeded TAD formation (Ke et al. 2017); (4) Specific chromatin interactions can facilitate further chromatin folding. A high-resolution computational method predicted that a small set of specific interactions is sufficient to drive chromatin folding in *Drosophila* embryos (Sun et al. 2021). It is worth noting that these chromatin structures are mainly composed of contacts between inactive regions and represent known long-range interactions with the biological function of gene silencing.

#### Phase separation

**Fig. 2:**
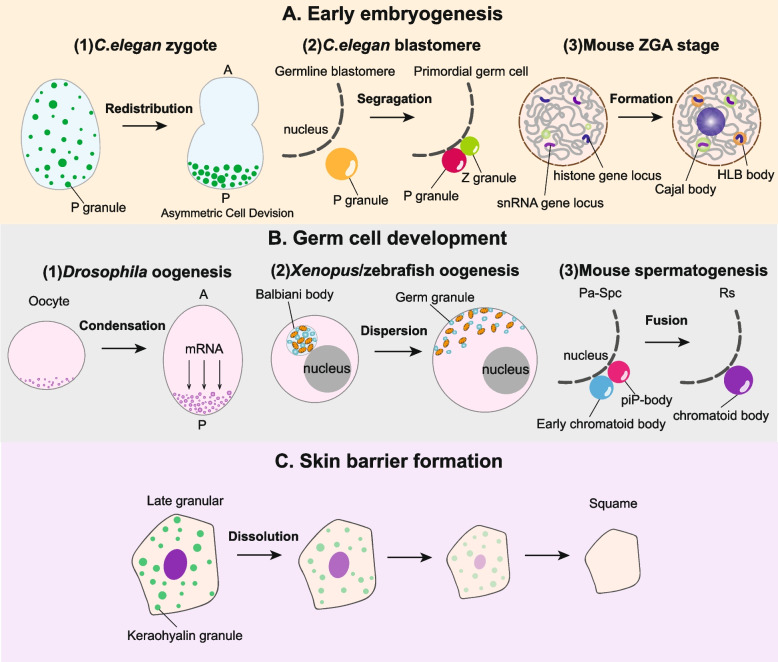
Dynamics of phase separation during different cell fate transitions. **A** Typical phase separation dynamics during early embryogenesis. (1) In *C.elegans* zygote, P granules are initially distributed in the cytoplasm evenly, and become enriched in the posterior by asymmetric cell division. (2) During *C.elegans* germline blastomere-to-germ cell transition, Z granule (green) separates from P granule (yellow) near the nucleus. (3) At the onset of ZGA in mice, nucleolus (purple), Cajal bodies (green) and HLB bodies (orange) are assembled at rRNA gene locus, snRNA gene locus and histone gene locus respectively. **B** Typical phase separation dynamics during cell differentiation. (1) During *Drosophila* oogenesis, polar granules aggregate at the posterior pole of oocytes initiated by mRNA transport from the nurse cells. (2) During *Xenopus*/zebrafish oogenesis, the Balbiani body is a large condensate consisting of germ granules and mitochondria near the nucleus, and disperses to vegetal hemisphere during oocyte growth. (3) During mouse spermatogenesis, piP-body locates adjacent to pi-bodies in prospermatogonia firstly, and then fuses with pro-chromatoid body to form mature chromatoid body before round spermatid stage. **C** During skin barrier formation, keratohyalin granules are gradually formed, and then dissolved due to the dramatic pH decrease

Germ granules are the earliest known and most studied phase-separated membrane-less organelles in germ cells during early embryonic development, such as P granules in *Caenorhabditis.elegans* and polar granules in *Drosophila* (Brangwynne et al. 2009; Trcek and Lehmann 2019). These condensates consist of RNAs and RNA-associated proteins and determine which regions of zygote will differentiate into germ cells. P granules are highly-dynamic liquid condensates distributed continuously in the germline of *C.elegans*. PGL-1 and PGL-3 form a core condensate of P granules to recruit other components. MEG-3 and MEG-4 are intrinsically disordered proteins that drive the formation of PGL condensates in the posterior of zygotes. P granules are initially evenly distributed in the cytoplasm of *C.elegan* zygotes and accumulate in the posterior (Fig. 2A) upon asymmetric division (Brangwynne et al. 2009). In addition, ZNFX-1 and WAGO-4 separate from P granules to form Z granules near the nucleus during germline blastomere-to-germ cell.

Nuclear bodies, such as nucleolus, Cajal bodies (CB), and histone locus bodies (HLBs), also undergo dynamics during early embryogenesis. Nucleolus is the nuclear compartment of rRNA transcription and processing, as well as ribosomal assembly (Lafontaine et al. 2021). Nucleolus formation at rDNA sites is dependent on the activation of rRNA transcription by RNA polymerase I in mice, zebrafish, *Drosophila,* and *C.elegans* embryos (Berry et al. 2015; Falahati et al. 2016; Zatsepina et al. 2003). Cajal bodies are composed of phase-separated protein coilin, snRNPs and snRNAs, wherein the snRNPs are assembled and snRNAs are modified (Machyna et al. 2013). At the onset of ZGA in zebrafish, CB assembly occurs on the snRNA gene locus (Heyn et al. 2017). Although many CBs in the zygote come from maternal and paternal pronuclei, transcriptional inhibition can decrease CB amount, suggesting that CB formation is partly dependent on snRNA transcription (Strzelecka et al. 2010). Similar to the nucleolus and CBs, HLBs do not mature at the histone gene loci until they are transcriptionally activated during ZGA (Heyn et al. 2017; Tatomer et al. 2016). Generally, nuclear bodies usually assemble at distinct genomic loci and are highly dependent on both proto-structures and transcriptional activation during early embryogenesis.

### Cell differentiation

#### Chromatin organization

Most studies on the dynamics of 3D chromatin structure have focused on stem cell differentiation (Bonev et al. 2017; Boya et al. 2017; Dixon et al. 2015; Zhang et al. 2019, 2020). Reprogramming of 3D chromatin is an elaborate process involving changes in chromatin hierarchical structures during cell differentiation. Embryonic stem cells (ESCs) have the ability of multi-directional differentiation and self-renewal, which corresponds to a highly plastic chromatin structure with decondensed heterochromatin (Dixon et al. 2015). FIREs are tissue-specific regions of high local interactions enriched with super-enhancers (Schmitt et al. 2016), and almost 60% of FIREs were detected in only two or fewer tissues and cell lines among 21 examined samples. Distinct 3D characteristics in different cell types are related to specific gene expression and biological functions closely. We have reviewed the characteristics of hierarchical chromatin structure dynamics during cell differentiation (Fig. 1B) in the following sections.

1. Compartment switching, including A-B and B-A transitions, frequently occurs consistent with gene expression and epigenetic changes dynamics during cell differentiation. Moreover, an increase of B compartments and interactions within B compartments have been observed within the respective datasets of different studies. Large extensions of heterochromatin appear in human ESCs during differentiation to mesenchymal stem cells (MSCs) and human embryonic lung fibroblasts (IMR90) (Dixon et al. 2015). During the late differentiation stages in mouse hematopoiesis, chromatin becomes more condensed, and long-range chromatin interactions are reduced (Zhang et al. 2020). Megakaryocyte-erythrocyte progenitor cells and granulocytes display obvious centromere clustering and telomere clustering resembling Rabl configuration, in which centromeres are clustered at one pole of the nucleus and telomeres are clustered on the opposite side during the interphase (Cowan et al. 2001; Duan et al. 2010; Stevens et al. 2017). Mouse neuron differentiation is also characterized by global chromatin compaction, with continuously increased interactions within B compartment and decreased interactions within A compartment (Bonev et al. 2017). Furthermore, A compartment is known to reduce by 5% during ESCs-to-neural progenitor cells (NPCs) transition (Dixon et al. 2015). Another study showed that human cardiomyocyte differentiation is accompanied by more packed heterochromatin and increased long-range intra-chromosome interaction in B compartment (Zhang et al. 2019).
2. The number of TAD boundaries decreases, and the average size of TADs increases during differentiation. Most TAD boundaries are highly conserved in different cell types (Schmitt et al. 2016). However, specific fractions of TAD boundaries disappear or emerge during cell differentiation. For instance, the total number of TADs decreases from 2008 to 1810 and average TAD size increases (from 800 kb to 920 kb) during pre-pro-B to pro-B cell transition (Boya et al. 2017). Similar changes have been observed during human cardiomyocyte differentiation and mouse neuronal differentiation (Bonev et al. 2017; Zhang et al. 2019). There are some mechanisms which may be associated with TAD changes during differentiation, such as the regulation of architectural factors, lineage-specific transcription factors, and epigenetic modifications. However, the exact molecular mechanisms underlying TAD reorganization remain to be elucidated.
3. Contacts of *cis*-regulatory elements are highly dynamic to regulate differentiation-associated gene expression. Enhancer-promoter or promoter-promoter interactions play an essential role in transcriptional regulation during cell differentiation. Extremely long-range promoter-promoter interactions are established during the transition from the 2i ground-state to the primed serum state of mouse ESCs, which implies that the initiation of ESC differentiation may be associated with these early-established interactions (Joshi et al. 2015). Furthermore, cell type-specific enhancer-promoter interactions are established and are concurrent to gene expression patterns during neuron, adipocyte, B cell differentiation, and limb morphogenesis (Bonev et al. 2017; Boya et al. 2017; Kragesteen et al. 2018; Siersbaek et al. 2017). For instance, olfactory receptor gene clusters make specific inter-chromosomal contacts, and associated-enhancers form a super-enhancer during mouse olfactory sensory neuron differentiation (Monahan et al. 2019). In mouse hematopoiesis, gene-associating domains of highly-expressed genes show high interactions within gene bodies (Zhang et al. 2020).
4. Germ cell differentiation has certain unique characteristics of 3D dynamics compared to others. A/B compartments gradually become weaker in late-stage growing oocytes. Polycomb-associating domains marked by H3K27me3 appear in full-grown oocytes and disappear during germinal vesicle breakdown (Du et al. 2020). However, compartments, TADs, and loops dissolve and then reappear during rhesus monkey and mouse pachytene spermatogenesis (Vara et al. 2019; Wang et al. 2019b).

#### Phase separation

The dynamics of membrane-less compartments contribute to cell identity and function during differentiation. Germ granules are diverse and cell type-specific and serve as excellent models for studying phase separation during germ cell development (Dodson and Kennedy 2020; So et al. 2021). Subcellular localization of germ granules may have important functions hitherto unknown in germ cell maturation. During mid-oogenesis in *Drosophila*, polar granules aggregate at the posterior pole of the oocytes (Fig. 2B), which is initiated by mRNA transport from the nurse cells (Trcek and Lehmann 2019). In *Xenopus* and zebrafish, the Balbiani bodies are first organized by germ granules and mitochondria near the nucleus and then disperse to vegetal hemisphere during oocyte growth (Bontems et al. 2009; Marlow and Mullins 2008; Schumacher et al. 2021). They contain germplasm that is essential for primordial germ cell formation, while its exact function is not fully understood. During mouse spermatogenesis, piP-body and pi-body are involved in the processing of pre-pachytene PIWI-interacting RNAs, a group of small RNAs that mediate transposon silencing. The piP-bodies often localize adjacent to pi-bodies in the prospermatogonia and fuse with pro-chromatoid bodies before the round spermatid stage to form mature chromatoid bodies (Aravin et al. 2009; Shoji et al. 2009). These findings indicate that the relative location of membraneless and membrane-bound organelles may be linked functionally and contribute to germ cell differentiation.

The transition from epidermal keratinocytes to squames is another typical process with dynamics of keratohyalin granules (KGs) (Quiroz et al. 2020). KGs gradually form from basal progenitors to granular cells and dissolve from late-granular cells to squamous cells due to the significant decrease of pH (Fig. 2C).

Like typical membrane-less organelles, some transcriptional regulators and epigenetic factors function in the form of protein-mediated phase separation during cell differentiation (Daneshvar et al. 2020; Kuang et al. 2021; Liu et al. 2020b). The evolutionarily conserved homeodomain transcription factor Prospero facilitates terminal neural differentiation of *Drosophila* neural precursors via LLPS on mitotic chromosomes, where it recruits and condenses HP1α to drive heterochromatin formation (Liu et al. 2020b). Likewise, the transcriptional coactivator SS18 regulates Brg/Brahma-associated factor complex through condensation to mediate pluripotent-somatic transition (PST) (Kuang et al. 2021). Furthermore, intrinsically disordered region (IDR) replacement of SS18 can rescue its primary function in PST.

In addition to protein-mediated phase separation, there are numerous RNA condensates consisting of coding or non-coding RNAs, which play important roles in regulating phase separation through nucleotide sequence, length, structure, modifications, and interactions (Roden and Gladfelter 2021). In the nucleus, hundreds of non-coding RNAs can form high-concentration territories and organize nuclear compartments to regulate RNA processing, heterochromatin assembly, and gene expression (Quinodoz et al. 2021). The lncRNA DIGIT, a conserved developmental regulator, controls endoderm differentiation by promoting BRD3 condensation at enhancers of endoderm transcriptional factors (Daneshvar et al. 2020).

Based on these findings, we conclude that some DNA-binding proteins regulate gene expression and promote cell differentiation via LLPS, which may be further promoted by non-coding RNAs.

### Somatic cell reprogramming

#### Chromatin organization

Somatic cell reprogramming is a process wherein mature differentiated cells transform into pluripotent precursors induced by several master factors, including Oct4, Sox2, Klf4, and c-Myc (Takahashi and Yamanaka 2006). The current consensus is that somatic cell reprogramming exhibits reverse 3D chromatin reorganization (Fig. 1C) compared to stem cell differentiation.

1. The proportion of A compartments slightly increases during somatic cell reprogramming. During the transition from mouse embryonic fibroblasts (MEFs) to induced pluripotency stem cells (iPSCs), 25% of the compartments show frequent switching, including 14% B-A switching, 5% A-B switching, and 6% unstable switching (Wang et al. 2021). Another study showed that the proportions of A and B compartments remain unchanged during B cells - iPSCs reprogramming (Stadhouders et al. 2018). It is worth mentioning that B-A compartments mainly contain early developmental genes while A-B compartments contain immune-associated genes (Stadhouders et al. 2018).
2. The number of TADs increases, and the median TAD size decreases during somatic cell reprogramming, which is evidently opposite to TAD changes seen during stem cell differentiation. TAD reorganization can be divided into TAD shift, fusion, and segregation (Wang et al. 2021). Only a minor proportion of TAD boundaries were found to be altered by in situ Hi-C, including a strong TAD boundary gained or lost near pluripotent genes *Sox2* and *Nanog*, respectively (Stadhouders et al. 2018).
3. Lineage-specific enhancer-promoter contacts are established or removed during cellular reprogramming. The 3D enhancer hubs, a set of highly-connected active enhancers, are reorganized and in contact with pluripotency genes to facilitate transcriptional activation (Di Giammartino et al. 2019; Di Stefano et al. 2020). Enhancer-promoter interactions near somatic genes disappear, but some interactions of NPCs around pluripotency genes still exist in the iPSC cells obtained from NPCs (Beagan et al. 2016). In general, chromatin loops are reorganized but not all of them are perfectly rewired during somatic cell reprogramming.

#### Phase separation

Phase separation is emerging as a new mechanism underlying the involvement of transcription factors in somatic cell reprogramming. Somatic cells undergo reprogramming to iPSCs following the transduction of four master transcription factors (Oct4, Sox2, Klf4, and c-Myc) (Takahashi and Yamanaka 2006). An elegant work has revealed that OCT4 phase separation promotes MEF-iPSC reprogramming by TAD reorganization (Wang et al. 2021), which is the first time that phase separation of pioneer factors has been shown to facilitate reprogramming. Interestingly, another study found that KLF4 can form condensates with DNA fragments via DNA-bridging rather than IDR, and fusion of KLF4 condensates is likely to establish long-range chromatin contacts and mediate pluripotency gene transcription during somatic cell reprogramming (Di Giammartino et al. 2019). It’s worth noting that there are hundreds of zinc finger proteins in the human genome, which might make bridging-induced phase separation like KLF4. Thus, transcription factors can regulate gene expression by phase separation during somatic reprogramming.

### Transdifferentiation and cell senescence

#### Chromatin organization

Cell transdifferentiation refers to the artificial reprogramming from one mature somatic cell type to another mature somatic cell type without undergoing a pluripotent state (Graf and Enver 2009). During cell transdifferentiation, cell-specific transcription factors play pivotal roles in coordinating cell-of-origin gene repression and lineage-specific gene activation (Dall’Agnese et al. 2019; Stik et al. 2020). For example, in fibroblast-myoblast conversion, myogenic master transcription factor orchestrates gene expression by driving significant chromatin interactions of *cis*-regulatory elements and altering insulated neighborhoods. Compartment dynamics are analyzed during lineage conversions from fibroblasts and immune cells (Ma et al. 2021). Contiguous compartment switchable regions are identified as chromatin-changing units during fibroblast-hepatocyte transdifferentiation. Specifically, pre-existing accessible chromatin in B-to-A compartment switchable sites existed before induction, which may allow the chromatin-binding of pioneer factor Foxa3. Foxa3 can facilitate epigenetic activation, chromatin interactions, and hepatic gene expression during transdifferentiation. Thus, 3D chromatin reorganization in mature somatic cell type transformation is drastic and is highly dependent on cell-specific transcription factors, especially TAD and loop dynamics.

Cell senescence is associated with various changes in 3D chromatin structures. Replicative cell senescence (RS) is a fundamental biological process occurred in aging, embryonic development, and tumor suppression (Liu et al. 2019). During RS, a small fraction of TADs containing 20% of the genes undergo compartment switching, and long-range contacts increase and short-range contacts decrease (Criscione et al. 2016). While in oncogene-induced senescence (OIS) cells, Lamin-associated domains (LADs) are lost and involved in the assembly of senescence-associated heterochromatin foci, which is accompanied by the dramatic loss of local interactions (Chandra et al. 2015).

#### Phase separation

During transdifferentiation, to date, there is still no published research reporting the dynamics of phase separation. Besides, during cell senescence, especially in OIS, researches mainly focused on the dynamics of senescence-associated heterochromatin foci (SAHF), which are specialized domains of facultative heterochromatin formed in senescent cells (Narita et al. 2003; Sati et al. 2020).

### Disease

#### Chromatin organization

Different from a normal physiological state, 3D chromatin architecture exhibits aberrant changes in many diseases, especially cancer, developmental disorders and cardiopathy (Bertero and Rosa-Garrido 2021; Boltsis et al. 2021; Li et al. 2018). Structural variations are common causes of TAD boundary disruption and abnormal chromatin loops (Spielmann et al. 2018). Studies are increasingly focusing on whether structural variations on non-coding DNA sequences cause aberrant pathogenic gene expression by changing 3D chromatin organization. A greater understanding of 3D chromatin disruption in diseases may provide new perspectives for clinical treatment. Here, we have reviewed 3D chromatin misfolding in cancer, developmental disorders, cardiac diseases, and other diseases (Fig. 1D).

##### Cancer

In cancer cells, 3D chromatin aberrations can occur in different hierarchical structures, and may have an impact on carcinogenesis. Nearly 12% of the genomic regions in breast cancer cell line MCF-7 display compartment switching compared to normal cell line MCF10A (Barutcu et al. 2015). Furthermore, A-B and B-A switching are respectively associated with downregulated and upregulated gene expression. Similar compartment changes could also be observed in multiple myeloma (MM) (Wu et al. 2017). The most studied 3D chromatin alterations in cancer cells are TAD boundary disruption and enhancer hijacking, which are closely associated with dysregulated gene expression (Flavahan et al. 2016; Groschel et al. 2014; Hnisz et al. 2016; Northcott et al. 2014). For instance, the total number of TAD boundaries is increased and the mean size of TADs is reduced in prostate cancer and multiple myeloma cells (Taberlay et al. 2016; Wu et al. 2017). TAD disorganization mainly results from two reasons: linear genomic variations as well as abnormal binding of structural factors due to epigenetic dynamics at TAD boundaries.

Malignant transformation of cells is accompanied by structural variations (SVs), including deletions, insertions, duplications, and translocations (Spielmann et al. 2018). Apart from gene dosage, structural variations have been proved to promote carcinogenesis by disruption of high-order chromatin structure (Dixon et al. 2018; Weischenfeldt et al. 2017). They can induce neo-TADs that encompass oncogenes, such as *MYC* and *ERBB2*, and even cause abnormal promoter-enhancer interactions and oncogene dysregulation. In T-cell acute lymphoblastic leukemia, microdeletions at TAD boundaries result in proto-oncogene activation, such as *TAL1* and *LMO2 *(Hnisz et al. 2016).

Abnormal structural factor binding on the genome is another cause of TAD and loop disorganization during cancer development. In glioma, hypermethylation at CTCF and cohesin binding sites leads to loss of CTCF- binding at a TAD boundary, which aberrantly activates of oncogene *PDGFRA* through constant interactions with its enhancer (Flavahan et al. 2016).

##### Developmental disorder

Disruption of high-order chromatin structure can cause developmental disorders, such as congenital limb malformation and cohesinopathies. Preaxial polydactyly is a common congenital hand disorder, which is attributable to *Shh* misexpression. *Shh* interacts with *ZRS*, a unique enhancer located 1 MB upstream from it (Lettice et al. 2003; Williamson et al. 2016)*.* Deletions of CTCF-binding sites around *ZRS* can lead to reduced interactions between *Shh* and *ZRS*, eventually resulting in deregulated *Shh* expression (Paliou et al. 2019)*.* Duplication of the *Sox9* regulatory region leads to the formation of a neo-TAD, which upregulates *Kcnj2* and results in limb malformation phenotype in mice (Franke et al. 2016). Femoral hypoplasia is closely associated with ectopic chromatin contacts rather than gene dosage effect at *FGF8* locus due to duplication (Franke et al. 2016). Therefore, ectopic interactions between enhancers and genes are archetypical genetic causes of developmental disorders. Cohesinopathies are another group of developmental diseases caused by mutations in the cohesin core and regulatory proteins (Bose and Gerton 2010). Knocking out bromodomain-containing protein 4 (BRD4) in the neural crest leads to decreased contact frequencies of chromatin loops and phenotypes similar to that seen in cohesinopathies (Linares-Saldana et al. 2021).

##### Cardiac disease

Studies increasingly show a correlation between 3D chromatin disruption and cardiac diseases. *LMNA* is one of the most frequently mutated genes in dilated cardiomyopathy (DCM) (Bertero et al. 2019; Lee et al. 2019b). *LMNA* mutations strengthened compartment segregation and altered the occupancy of Lamin-associated domains (LADs) in haplo-insufficient human iPSC models of *LMNA-*related DCM. Redistributed LADs were associated with increased CpG methylation and gene repression, although A/B compartment switching occurred in only 1% of the genome (Bertero et al. 2019). Heart failure is a severe cardiac disease accompanied by dramatic 3D changes. CTCF is downregulated in patients with heart failure, and deletion of *Ctcf* in mice leads to heart failure and 3D chromatin reorganization (Lee et al. 2019a; Rosa-Garrido et al. 2017), including 99% loss of chromatin loops, TAD disruption, and A/B compartment switching in nearly 4% of the genome. Furthermore, ectopic long-range interactions between cis-regulatory elements in 4q25 and promoters of *Pitx2c* and *Enpep* may be an indirect genetic risk for fibrillation (Aguirre et al. 2015).

##### Other diseases

Laminopathies, autoimmune diseases, and infectious diseases are also associated with 3D chromatin alterations. Laminopathies encompass a wide range of genetic disorders resulting from over 400 mutations, the majority of which are linked with *LMNA *(Shin and Worman 2022). Hutchinson-Gilford Progeria Syndrome (HGPS) is the most characteristic progeroid laminopathy caused by a de novo point mutation at position 1824 of *LMNA*. In HGPS fibroblast cells, a subset (12%) of compartments eventually undergoes switching (Chandra et al. 2015), and chromatin compartmentalization strength and Lamin A/C-heterochromatin interactions are globally reduced compared to that in the normal cell line. Capture Hi-C also showed for the first time that the risk loci of rheumatoid arthritis have strong contacts with the promoter of gene *AZI2* in NF-κB pathway in immune cells (Martin et al. 2015).

#### Phase separation

Neurodegenerative disorders and cancer are two main diseases related to phase separation (Spannl et al. 2019). Aberrant aggregation of disease-related proteins or disruption of some functional phase separation can lead to pathological changes. Insoluble protein aggregation is the most common pathological phenotype in neurodegenerative diseases, including Tau aggregation in Alzheimer’s disease (Jucker and Walker 2013), Lewy body in Parkinson’ s disease (Polymeropoulos et al. 1997; Spillantini et al. 1997), huntingtin exon1 aggregation in Huntingtin disease (Peskett et al. 2018), and stress granule protein aggregation in amyotrophic lateral sclerosis and frontotemporal dementia (Aulas and Vande Velde 2015; Elbaum-Garfinkle 2019). Phase separation disruption or changes in location, components, or condensation caused by genetic mutations can drive cancer development (Boija et al. 2021). For example, chromatin condensates and gene expression are disrupted due to the histone mutation H3K27M and H3K36M respectively occurred in brainstem gliomas and sarcoma (Larson et al. 2019; Lu et al. 2016). In acute lymphoblastic leukemia, transcriptional condensate mislocalization due to SVs can lead to oncogene activation (Mansour et al. 2014). Some congenital hereditary diseases are also associated with abnormal transcriptional condensates. Repeat expansions in transcription factors alter their phase separation ability and perturb their transcriptional condensates in a mouse model of synpolydactyly (Basu et al. 2020). Likewise, MLL4-associated transcriptional condensates are disrupted, and nuclear mechanical stress mediated by PcG bodies increases in the Kabuki syndrome disease model (Fasciani et al. 2020). Rett syndrome-associated mutations in MeCP2 reduce its phase separation ability to form heterochromatin condensates, which may contribute to Rett syndrome pathogenesis (Wang et al. 2020). In addition, in the skin barrier disorders, altered phase separation dynamics of KGs are caused by filaggrin mutations or environmental changes (Quiroz et al. 2020).

### Brief summary

In conclusion, 3D chromatin misfolding is not only a set of accompanying dynamics, but also acts as a key cause in some diseases. High-order chromatin structure rewiring in diseases can occur due to the following reasons: (1) Point mutations or structural variations in enhancers or target genes, (2) structural variations at chromatin structural factor-binding sites, and (3) mutations of chromatin structural factors or other regulatory proteins. The underlying mechanisms of compartment switching in disease are still not fully understood. Phase separation has been proposed to facilitate compartment formation (Larson et al. 2017; Strom et al. 2017; Zenk et al. 2021), and is likely to be involved in abnormal compartment changes (Wang et al. 2020). Further studies are needed to understand how 3D chromatin dynamics affect the pathogenesis of critical diseases in order to devise novel therapeutic strategies.

The behaviors of phase separation may have possible functional effects on cellular activities. It has been assumed that phase-separated compartments may promote the efficiency of biological process by increasing local concentration and interactions of distinct components. Besides, they may buffer the average concentration of a given component within the cells (Bergeron-Sandoval et al. 2016). More investigations should be put into the possible functions of phase separation.

## Phase separation is involved in 3D chromatin reorganization

There is ample evidence that nuclear phase separation can directly regulate 3D chromatin reorganization (Ahn et al. 2021; Liu et al. 2021; Shi et al. 2021; Shin et al. 2019; Wang et al. 2021). However, the exact role of phase separation in 3D chromatin reorganization has not been completely elucidated in most studies. We have summarized the direct and indirect evidence of the relationship between phase separation and 3D chromatin reorganization in the following sections.

### Direct evidence that phase separation can facilitate 3D chromatin reorganization

**Fig. 3:**
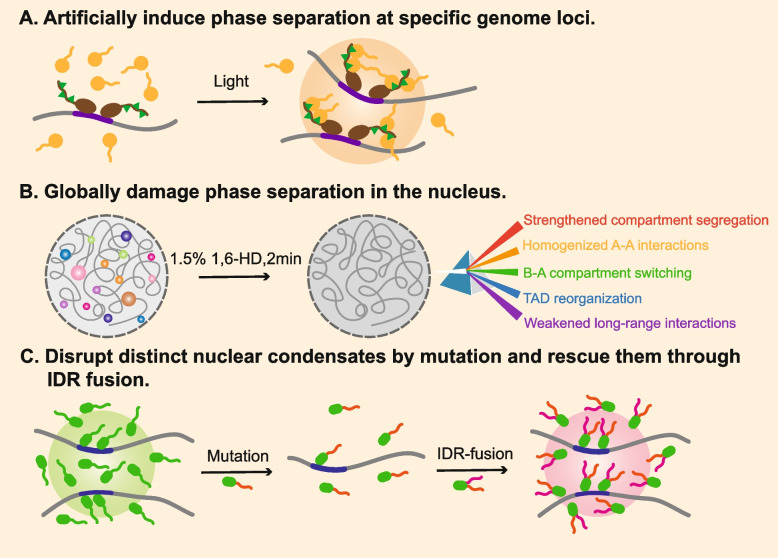
Research strategies for studying the relationship between 3D chromatin organization and phase separation. **A** Artificially induce condensates at specific genomic loci to form chromatin loops by the combination of light induction, CRISPR-Cas9, and IDR-induced phase separation in CasDrop system. **B** Globally damage phase separation in the nucleus by 1.5% 1,6-HD treatment for 2 min, which leads to strengthened compartment segregation, homogenized A-A interactions, B-A compartment switching, TAD reorganization and weakened long-range interactions. **C** Disrupt distinct nuclear condensates by key phase-separating protein mutations and rescue them through IDR fusion to observe 3D chromatin reorganization

The relationship between phase separation and 3D chromatin reorganization can be analyzed by three research strategies: (1) artificially induce liquid condensates at specific genomic loci to observe 3D chromatin alterations (Fig. 3A), (2) global damage of phase separation to observe changes in global chromatin structures (Fig. 3B), and (3) disruption of distinct nuclear condensates by mutating key factors and rescuing through IDR fusion (Fig. 3C).

The most forceful method currently is the CasDrop system, which uses liquid condensation to restructure chromatin loops (Shin et al. 2019). Based on CRISPR-Cas9 and optogenetic technology, CasDrop can pull targeted genomic loci together via IDR-driven condensates. Intriguingly, the droplets tend to grow in chromatin regions of low density and mechanically exclude heterochromatin, which suggests that liquid condensates can construct chromatin contacts selectively through “chromatin filtering”. Disrupting and rescuing phase separation globally or specifically is another effective way to directly study its relationship with 3D chromatin reorganization (Liu et al. 2021). 1,6-hexanediol (1,6-HD) is a useful chemical that can dissolve liquid condensation by disrupting weak hydrophobic protein-protein or protein-RNA interactions (Cermakova and Hodges 2018; Kroschwald et al. 2017), while improper use of 1,6-HD (high concentration and long term) can result in the loss of membrane integrity, cell shrinkage and aberrant aggregation of proteins. 1,5% 1,6-HD for 2 min was found to be the optimal using condition to dissolve phase separation in living cells (Liu et al. 2021). Treatment of 1.5% 1,6-HD for 2 min in mESCs leads to 3D chromatin reorganization at different hierarchies, including strengthened A/B compartment segregation, homogenized A-A interactions, B-A compartment switching, TAD reorganization, and weakened long-range interactions. These remarkable changes indicate that phase separation is a widespread state for numerous molecules to maintain stable 3D chromatin organization.

Different from global phase separation damage in the nucleus, interference with distinct nuclear biomolecular condensates only causes the rewiring of more refined high-order chromatin structures. Phase separation of master transcription factors can directly regulate 3D chromatin reorganization. Disruption of OCT4 phase separation via acidic mutations attenuates TAD reorganization and somatic cell reprogramming, which can be rescued by fusing FUS-IDR to OCT4 (Wang et al. 2021). In the OCT4 phase, the formation of inter-TAD OCT4 loops may drive neighboring TAD fusion. In addition, CTCF mediates long-range chromatin interactions between A compartments through RING1 and YY1 binding protein (RYBP) -dependent phase separation (Wei et al. 2022). Induced CTCF phase separation can restore inter-A interactions after RYBP depletion, which provides a new insight into the mechanisms of how structural factors facilitate chromatin loops apart from loop extrusion. KLF4 is thought to induce long-range pluripotency contacts through condensation at promoters of pluripotency factors (Sharma et al. 2021). However, KLF4 condensation depends on KLF4-DNA bridging not IDR, indicating that zing-finger proteins may contribute to chromatin contacts via DNA-binding-mediated phase separation. More in vivo assays are needed to confirm the importance of KLF4 phase separation in 3D chromatin organization and cell fate regulation.

Aberrant protein phase separation and the ensuing 3D chromatin misfolding can lead to cancer. For example, phase separation of protein chimera NUP98-HOXA9 at proto-oncogenes in human leukemia induced CTCF-independent chromatin loops and promoted carcinogenesis (Ahn et al. 2021). In addition, loss of liquid condensation also leads to tumor development. UTX is a key tumor suppressor with a strong phase-separating ability, and its IDR-lacking mutant eliminated condensation and resulted in the loss or gain of long-range chromatin loops in the acute myeloid leukemia (AML) cell line (Shi et al. 2021). Therefore, changes in liquid condensation propensity in proteins can promote tumorigenesis through aberrant long-range interactions.

### Indirect evidence of possible regulatory roles of phase separation in 3D chromatin reorganization

Most studies’ evidence correlating phase separation with 3D chromatin organization is indirect. On the one hand, many nuclear condensates are shown to regulate 3D chromatin organization, such as the nucleus, nuclear speckles, Cajal body, PcG body, and BRD4 condensates (Bantignies and Cavalli 2011; Linares-Saldana et al. 2021; Quinodoz et al. 2018; Sawyer et al. 2016a, b; Schoenfelder et al. 2015; Wang et al. 2016a). On the other hand, some chromatin structural factors and heterochromatin-related proteins have the ability to form LLPS (Larson et al. 2017; Ryu et al. 2021; Sanulli et al. 2019; Strom et al. 2017; Wang et al. 2020, 2022).

#### Nuclear condensates are involved in 3D chromatin organization

Nuclear bodies recruit specific genomic loci to their peripheries and regulate gene expression. Chromatin interactions, especially gene clusters, are predominantly observed around these subcellular structures. As the most common bodies in the nucleus, both nucleolus and nuclear speckles contribute to the organization of high-order chromatin interactions. A large number of inter-chromosome interactions have been identified by SPRITE, and divided into active and repressive hubs based on gene density and transcription (Quinodoz et al. 2018). Repressive hubs preferentially form around the nucleolus, while active hubs are arranged near nuclear speckles. Furthermore, disruption of nuclear speckles by *Srrm2* knockdown specially reduces the insulator score of TADs in active compartments (Hu et al. 2019).

Cajal bodies form and maintain a number of intra-chromosomal and inter-chromosomal gene clusters detected by 4C-seq and DNA FISH technique (Sawyer et al. 2016b, Wang et al. 2016a). The transcriptionally active spliceosomal U snRNA/snoRNA genes and histone genes are in close proximity to the CBs, and the disassembly of CBs disrupts these gene clusters and inhibits snRNA/snoRNA and histone gene expression.

PcG bodies organize repressive genome interaction network to regulate gene silencing functionally. Polycomb repressive complex 1 (PRC1) protein CBX2 is able to undergo phase separation and may be the driver for PRC1 to form liquid condensates (Tatavosian et al. 2019). Furthermore, the propensity of LLPS also contributes to targeted H3K27me3-marked chromatin organization. Long-range chromatin interactions have been detected between PcG-repressed regions, such as *Hox* gene clusters (Bantignies et al. 2011; Schoenfelder et al. 2015; Sexton et al. 2012). In *Drosophila*，“*Hox* gene kissing” is an intriguing phenomenon referring to contacts between two repressed *Hox* gene clusters (Antennapedia complex and bithorax complex, respectively) within PcG bodies (Bantignies et al. 2011; Sexton et al. 2012). In mouse ESCs, PRC1 functions as a key regulator of 3D chromatin organization by mediating gene network, and the strongest contacts consist of 4 *Hox* gene clusters and early developmental transcription factors (Schoenfelder et al. 2015). PRC1 knockout causes disruption of promoter-promoter contacts. Interactions between poised enhancers and promoters facilitate neural induction in a PRC2-dependent manner during ESC differentiation (Cruz-Molina et al. 2017). Thus, PcG bodies are a key organizer of chromatin-interacting network and gene repression to facilitate cell fate transition.

A few components of transcriptional machinery, such as RNA Pol II, Mediator, and BRD4, contain IDRs and are capable of forming liquid condensation (Boehning et al. 2018; Boija et al. 2018; Cho et al. 2018; Nagulapalli et al. 2016; Sabari et al. 2018), which may be involved in the 3D chromatin folding. BRD4 degron leads to decreased NIPBL occupancy as well as contact frequencies of most chromatin loops (*n* = 5298/7517), suggesting that BRD4 may be involved in 3D chromatin folding through liquid-liquid phase separation (Linares-Saldana et al. 2021). Functional transcription is regulated by transcription factor residence time, multivalent interactions, and phase separation. It has been proposed that multivalent interactions of activating domains are sufficient to enhance transcription. Liquid droplets only increase local TF concentration, but cannot enhance the activation of transcription (Trojanowski et al. 2022).

#### Chromatin structural factors can undergo phase separation

Polymer-polymer phase separation and liquid-liquid phase separation have been proposed as essential mechanisms for chromatin structural factors to construct the 3D chromatin architecture (Erdel and Rippe 2018). Structural maintenance of chromosome (SMC) protein complex induces loop extrusion and is crucial for the establishment and maintenance of chromatin loops (Davidson and Peters 2021). Yeast SMC protein can form liquid-like condensates with DNA through DNA-bridging in vivo, and the cohesin-DNA clustering is strongly dependent on the DNA length in vitro (Ryu et al. 2021). Furthermore, computational modeling based on polymer physics has shown that polymer phase separation of chromatin structure factors including cohesin and CTCF, is a key molecular mechanism regulating 3D chromatin organization at the single molecular level (Conte et al. 2020). YY1, another structural factor of enhancer-promoter looping, can form LLPS by the histidine cluster to coordinate coactivators and activate gene expression (Wang et al. 2022).

#### Heterochromatin related proteins or ncRNAs drive chromatin compartmentalization in the form of phase separation

Chromatin undergoes intrinsic LLPS in physiological salt modulated by histone H1, linker DNA length, and histone acetylation (Gibson et al. 2019). Several investigations have shown that heterochromatin related proteins are essential for heterochromatin compaction through phase separation and promote compartmentalization by mediating heterochromatin interactions (Larson et al. 2017; Strom et al. 2017; Wang et al. 2020; Zenk et al. 2021). HP1α is the best-known binding protein of transcription-silencing chromatin region marked by H3K9 methylation and can induce chromatin into droplet-like condensation in vivo (Larson et al. 2017; Strom et al. 2017). The driving forces of HP1α phase separation are multivalent H3K9me3-chromodomain interactions and increasing dynamics within histone octamer core (Sanulli et al. 2019; Wang et al. 2019a). In contrast, another study showed that HP1α has a limited ability to form liquid droplets in mouse fibroblasts and chromocenter is maintained independently of HP1α LLPS (Erdel et al. 2020). MeCP2 is a ubiquitous binding partner to DNA methylation and plays an important role in transcriptional repression (Jones et al. 1998; Nan et al. 1998). Similar to HP1α, MeCP2 can induce LLPS of nucleosomal arrays in vitro as well (Wang et al. 2020). Pathological mutations of MeCP2 compromise MeCP2-associated chromatin condensation in Rett syndrome. Nevertheless, we still lack a complete understanding of the biophysical basis of heterochromatin compaction, and the extent to which phase separation contributes to heterochromatin formation in vivo requires a more exact answer.

The ncRNA *Xist* can induce X-chromosome inactivation by recruiting repressive protein complexes to chromatin (Chu et al. 2015; McHugh et al. 2015). Notably, *Xist* foci spreading along the X chromosome are actually phase-separated condensates dependent on multivalent E-repeat elements of *Xist* and self-aggregation of *Xist*-binding proteins (Jachowicz et al. 2022; Pandya-Jones et al. 2020). Hence, phase separation drives the formation of *Xist* loci and ensures the persistent inactivation of X chromosome.

#### Chromatin models based on polymer phase separation can reconstruct the 3D chromatin organization

Computational methods have provided evidence that phase separation can serve as a new mechanism of chromatin folding beyond loop extrusion (Conte et al. 2020; Esposito et al. 2022). Polymer models have been developed to make predictions on contacts between distal DNA binding sites by soluble molecular factors (e.g., transcription factors), thermodynamic mechanisms of phase separation or interaction probabilities based on diffusional motion (Bohn and Heermann 2010; Brackley et al. 2013, 2016a, b; Chiariello et al. 2016, 2020; Conte et al. 2020; Di Pierro et al. 2016; Esposito et al. 2022; Nicodemi and Prisco 2009). Based on polymer model and machine learning from Hi-C bulk data, a chromatin model can reconstruct 3D chromatin structure consistent with single-cell super-resolution microscopy results, revealing that polymer phase separation is likely to drive the 3D chromatin conformation (Conte et al. 2020). In another study, polymer physics is sufficient to recapitulate 3D chromatin contact patterns across the entire genome (Esposito et al. 2022). Importantly, the combinatorial action of epigenetic factors seems to be important to explain the complex contact patterns.

### Brief summary

Summing up, phase separation is emerging as an important mechanism involved in 3D chromatin organization at different hierarchies. OCT4 and CTCF-interactors phase separation are two compelling examples facilitating 3D chromatin structure directly. Typical nuclear bodies and local condensation of different chromatin-binding factors, including transcriptional factories, structural factors, heterochromatin related proteins and ncRNAs, are likely to be involved in chromatin folding through LLPS or PPPS. Apart from the above nuclear condensates, other known condensates (e.g., paraspeckles and PML nuclear bodies) may also regulate 3D chromatin organization in a similar way. More direct evidence is required to validate the causal relationship between phase separation and 3D chromatin organization.

## Discussion

Cell fate transitions are accompanied by dynamics of 3D chromatin organization and phase separation, and nuclear condensates can directly facilitate 3D chromatin organization. However, the complexity of chromatin folding is far beyond imagination since different regulatory mechanisms exist at the same time. There is a long-standing controversy over the functional roles of phase separation in enhancer-promoter interaction establishment and maintenance. By regulating cell type-specific gene expression, phase separation and 3D chromatin organization can concomitantly or independently affect cell fate transitions.

### The mechanisms of chromatin folding distinct from phase separation

Phase separations, including LLPS and PPPS, provide new insights into how chromatin is folded into complicated but mostly distinct structures at different hierarchies in the nucleus. Loop extrusion is the typical mechanism directly mediating chromatin looping by SMC complexes and CTCF. There are probably some unknown possibilities to explain chromatin folding, since cellular condensation can result from other mechanisms distinct from phase separation. One study found that recruitment of RNA Pol II and other factors to replication compartments is predominantly dependent on transient and non-specific binding to DNA during viral infection (McSwiggen et al. 2019). Therefore, phase separation is emerging as an important but not the only mechanism involved in 3D chromatin organization. In order to distinguish between phase separation and other possible mechanisms, compelling evidence based on quantitative experiments or other new strategies are undoubtedly required.

### Distinct mechanisms can underly E-P interaction establishment and maintenance

Specific enhancer-promoter interactions determine cell type-specific gene expression as well as cell fate transitions. There is some controversy regarding the regulatory mechanisms in the establishment and maintenance of E-P interactions. Loop extrusion had been thought of as a key controller of E-P interactions (Symmons et al. 2014). However, recent studies found that cohesin and CTCF are not required for E-P interactions through acute degradation or deletion of CTCF motifs (Aljahani et al. 2022; Chakraborty 2022). Furthermore, CTCF and a portion of cohesin in yeast interact with interactors or chromatin through phase separation (Ryu et al. 2021; Wei et al. 2022). RNA Pol II, transcription factors and cofactors with IDRs have a general property to assemble into enhancers and promoters through phase separation (Ahn et al. 2021; Boija et al. 2018; Cho et al. 2018; Sabari et al. 2018). However, rapid degradation of the phase-separated proteins, including Mediator, BRD4, Pol II, or YY1, separately has little impact on E-P contact frequencies (Crump et al. 2021; El Khattabi et al. 2019). Altogether, it seems that disrupting loop extrusion or phase separation cannot eliminate the transient maintenance of E-P interactions. The possible speculations are: (1) loop extrusion or phase separation mainly act at the establishment of E-P interactions. E-P interactions could be affected through at least one cell cycle, since E-P interactions go through re-establishing during cell cycle. (2) the underlying proteins which drive E-P interactions may display a great deal of redundancy. Removing one protein individually does not affect other proteins. (3) the role of these factors in regulating E-P interactions has the loci specificity of cell type-specific characteristics. Notably, a very recent study based on polymer physic model showed that loop extrusion and phase separation can co-exist simultaneously at the single-molecule level to recapitulate chromatin structure from Hi-C and microscopy data (Conte et al. 2022). In summary, there are probably different mechanisms sustaining functional E-P interactions at the same time, and it seems difficult to estimate which one is more vital across the whole genome by existing methods. Therefore, available methods should be explored. In addition, how E-P interactions are established is another key question remained to be answered.

### Coordinated or independent roles of 3D chromatin organization and phase separation in cell fate regulation

**Fig. 4:**
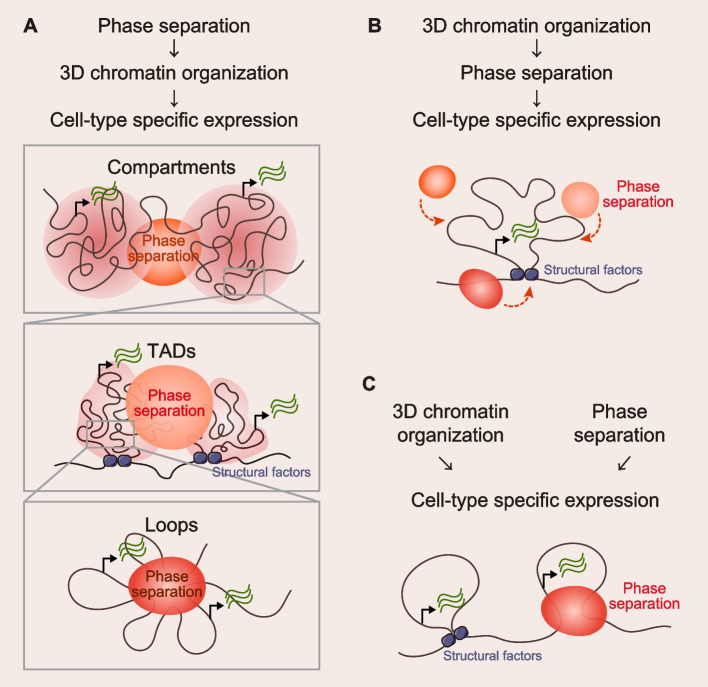
Coordinated or independent roles of 3D chromatin organization and phase separation in cell fate regulation. **A** Phase separation affects cell type-specific gene expression to regulate cell fate by regulating 3D chromatin structure at different hierarchies. **B** Specific chromatin structure recruits different phase separations and affects cell type-specific gene expression to regulate cell fate. **C** Phase separation and 3D chromatin structure affect cell type-specific gene expression to regulate cell fate without interfering with each other

The hallmark and key to cell fate transition is cell type-specific gene expression. Here we propose three possible models of 3D chromatin structure and phase separation in cell fate determination (Fig. 4): (1) Phase separation affects cell type-specific gene expression to regulate cell fate by regulating 3D chromatin structure at different hierarchies; (2) Specific chromatin structure recruits different phase separations, affects cell type-specific gene expression to regulate cell fate; (3) Phase separation and 3D chromatin structure affect cell type-specific gene expression to regulate cell fate without interfering with each other. These three models may coexist at different sites in cells, and more direct evidence is needed to explore and prove them.

## Conclusions and perspectives

There are still many enigmatic questions remained to be answered. How nuclear condensates (nuclear speckles, heterochromatin loci, transcriptional condensates and so on) are organized exactly? What molecular events related to 3D chromatin organization happen in or around these condensates in vivo? How do these nuclear condensates change accompanied with 3D chromatin reorganization during cell fate transitions?

More and more studies are focusing on whether phase separation is involved in 3D chromatin regulation currently, but it remains to be determined as to why regulators undergo LLPS to exert their functions. The possible functions of LLPS include to facilitate cellular activities by sequestering distinctive molecules and forming relatively-independent microenvironments, and to buffer the effective condensation of other cellular molecules. Further investigations are needed to elucidate the role of 3D chromatin organization in phase separation modulation, and new methods have to be developed to unravel the relationship between 3D chromatin and phase separation.

## Data Availability

Not applicable.

## Abbreviations

1,6-HD: 1,6-hexanediol
3D: Three-dimensional
CTCF: CCCTC-Binding Factor
DCM: Dilated cardiomyopathy
ESCs: Embryonic stem cells
FIREs: Frequently interaction regions
HP1α: Heterochromatin protein 1α
IDR: Intrinsically disordered region
iPSCs: Induced pluripotency stem cells
KGs: Keratohyalin granules
LADs: Lamin-associated domains
LLPS: Liquid-liquid phase separation
MeCP2: Methyl-CpG-binding protein 2
MM: Multiple myeloma
NPCs: Neural progenitor cells
OIS: Oncogene-induced senescence
PRC1: Polycomb repressive complex 1
PST: pluripotent-somatic transition
RS: Replicative cell senescence
SAHF: Senescence-associated heterochromatin foci
SMC: Structural maintenance of chromosome
SPRITE: Split-pool recognition of interactions by tag extension
TAD: Topologically associating domain
ZGA: Zygotic genome activation

## References

[CR1] Aguirre LA, Alonso ME, Badia-Careaga C, Rollan I, Arias C, Fernandez-Minan A (2015). Long-range regulatory interactions at the 4q25 atrial fibrillation risk locus involve PITX2c and ENPEP. BMC Biol.

[CR2] Ahn JH, Davis ES, Daugird TA, Zhao S, Quiroga IY, Uryu H (2021). Phase separation drives aberrant chromatin looping and cancer development. Nature.

[CR3] Aljahani A, Hua P, Karpinska MA, Quililan K, Davies JOJ, Oudelaar AM (2022). Analysis of sub-kilobase chromatin topology reveals nano-scale regulatory interactions with variable dependence on cohesin and CTCF. Nat Commun.

[CR4] Aravin AA, van der Heijden GW, Castañeda J, Vagin VV, Hannon GJ, Bortvin A (2009). Cytoplasmic compartmentalization of the fetal piRNA pathway in mice. PLoS Genet.

[CR5] Atlasi Y, Stunnenberg HG (2017). The interplay of epigenetic marks during stem cell differentiation and development. Nat Rev Genet.

[CR6] Aulas A, Vande Velde C (2015). Alterations in stress granule dynamics driven by TDP-43 and FUS: a link to pathological inclusions in ALS?. Front Cell Neurosci.

[CR7] Banani SF, Lee HO, Hyman AA, Rosen MK (2017). Biomolecular condensates: organizers of cellular biochemistry. Nat Rev Mol Cell Biol.

[CR8] Bantignies F, Cavalli G (2011). Polycomb group proteins: repression in 3D. Trends Genet.

[CR9] Bantignies F, Roure V, Comet I, Leblanc B, Schuettengruber B, Bonnet J (2011). Polycomb-dependent regulatory contacts between distant Hox loci in Drosophila. Cell.

[CR10] Barutcu AR, Lajoie BR, McCord RP, Tye CE, Hong D, Messier TL (2015). Chromatin interaction analysis reveals changes in small chromosome and telomere clustering between epithelial and breast cancer cells. Genome Biol.

[CR11] Basu S, Mackowiak SD, Niskanen H, Knezevic D, Asimi V, Grosswendt S (2020). Unblending of transcriptional condensates in human repeat expansion disease. Cell.

[CR12] Beagan JA, Gilgenast TG, Kim J, Plona Z, Norton HK, Hu G (2016). Local genome topology can exhibit an incompletely rewired 3D-folding state during somatic cell reprogramming. Cell Stem Cell.

[CR13] Beagrie RA, Scialdone A, Schueler M, Kraemer DC, Chotalia M, Xie SQ (2017). Complex multi-enhancer contacts captured by genome architecture mapping. Nature.

[CR14] Bergeron-Sandoval LP, Safaee N, Michnick SW (2016). Mechanisms and consequences of macromolecular phase separation. Cell.

[CR15] Berry J, Weber SC, Vaidya N, Haataja M, Brangwynne CP (2015). RNA transcription modulates phase transition-driven nuclear body assembly. Proc Natl Acad Sci U S A.

[CR16] Bertero A, Fields PA, Smith AST, Leonard A, Beussman K, Sniadecki NJ (2019). Chromatin compartment dynamics in a haploinsufficient model of cardiac laminopathy. J Cell Biol.

[CR17] Bertero A, Rosa-Garrido M (2021). Three-dimensional chromatin organization in cardiac development and disease. J Mol Cell Cardiol.

[CR18] Bintu B, Mateo LJ, Su JH, Sinnott-Armstrong NA, Parker M, Kinrot S, et al. Super-resolution chromatin tracing reveals domains and cooperative interactions in single cells. Science. 2018;362(6413). 10.1126/science.aau1783. 10.1126/science.aau1783 PMC6535145 30361340

[CR19] Boehning M, Dugast-Darzacq C, Rankovic M, Hansen AS, Yu T, Marie-Nelly H (2018). RNA polymerase II clustering through carboxy-terminal domain phase separation. Nat Struct Mol Biol.

[CR20] Bohn M, Heermann DW (2010). Diffusion-driven looping provides a consistent framework for chromatin organization. PLoS One.

[CR21] Boija A, Klein IA, Sabari BR, Dall’Agnese A, Coffey EL, Zamudio AV (2018). Transcription factors activate genes through the phase-separation capacity of their activation domains. Cell.

[CR22] Boija A, Klein IA, Young RA (2021). Biomolecular condensates and cancer. Cancer Cell.

[CR23] Boltsis I, Grosveld F, Giraud G, Kolovos P (2021). Chromatin conformation in development and disease. Front Cell Dev Biol.

[CR24] Bonev B, Mendelson Cohen N, Szabo Q, Fritsch L, Papadopoulos GL, Lubling Y (2017). Multiscale 3D genome rewiring during mouse neural development. Cell.

[CR25] Bontems F, Stein A, Marlow F, Lyautey J, Gupta T, Mullins MC (2009). Bucky ball organizes germ plasm assembly in zebrafish. Curr Biol.

[CR26] Bose T, Gerton JL (2010). Cohesinopathies, gene expression, and chromatin organization. J Cell Biol.

[CR27] Boya R, Yadavalli AD, Nikhat S, Kurukuti S, Palakodeti D, Pongubala JMR (2017). Developmentally regulated higher-order chromatin interactions orchestrate B cell fate commitment. Nucleic Acids Res.

[CR28] Brackley CA, Brown JM, Waithe D, Babbs C, Davies J, Hughes JR, et al. Predicting the three-dimensional folding of cis-regulatory regions in mammalian genomes using bioinformatic data and polymer models. Genome Biol. 2016a;17(1). 10.1186/s13059-016-0909-0. 10.1186/s13059-016-0909-0 PMC4815170 27036497

[CR29] Brackley CA, Johnson J, Kelly S, Cook PR, Marenduzzo D (2016). Simulated binding of transcription factors to active and inactive regions folds human chromosomes into loops, rosettes and topological domains. Nucleic Acids Res.

[CR30] Brackley CA, Taylor S, Papantonis A, Cook PR, Marenduzzo D (2013). Nonspecific bridging-induced attraction drives clustering of DNA-binding proteins and genome organization. Proc Natl Acad Sci U S A.

[CR31] Brangwynne CP, Eckmann CR, Courson DS, Rybarska A, Hoege C, Gharakhani J (2009). Germline P granules are liquid droplets that localize by controlled dissolution/condensation. Science.

[CR32] Cermakova K, Hodges HC. Next-generation drugs and probes for chromatin biology: from targeted protein degradation to phase separation. Molecules. 2018;23(8). 10.3390/molecules23081958. 10.3390/molecules23081958 PMC6102721 30082609

[CR33] Chakraborty. High affinity enhancer-promoter interactions can bypass CTCF/cohesin-mediated insulation and contribute to phenotypic robustness. bioRxiv. 2022. 10.1101/2021.12.30.474562.

[CR34] Chandra T, Ewels PA, Schoenfelder S, Furlan-Magaril M, Wingett SW, Kirschner K (2015). Global reorganization of the nuclear landscape in senescent cells. Cell Rep.

[CR35] Chen X, Ke Y, Wu K, Zhao H, Sun Y, Gao L (2019). Key role for CTCF in establishing chromatin structure in human embryos. Nature.

[CR36] Chiariello AM, Annunziatella C, Bianco S, Esposito A, Nicodemi M (2016). Polymer physics of chromosome large-scale 3D organisation. Sci Rep.

[CR37] Chiariello AM, Bianco S, Oudelaar AM, Esposito A, Annunziatella C, Fiorillo L (2020). A dynamic folded hairpin conformation is associated with alpha-globin activation in erythroid cells. Cell Rep.

[CR38] Cho WK, Spille JH, Hecht M, Lee C, Li C, Grube V (2018). Mediator and RNA polymerase II clusters associate in transcription-dependent condensates. Science.

[CR39] Chu C, Zhang QC, da Rocha ST, Flynn RA, Bharadwaj M, Calabrese JM (2015). Systematic discovery of Xist RNA binding proteins. Cell.

[CR40] Conte M, Fiorillo L, Bianco S, Chiariello AM, Esposito A, Nicodemi M (2020). Polymer physics indicates chromatin folding variability across single-cells results from state degeneracy in phase separation. Nat Commun.

[CR41] Conte M, Irani E, Chiariello AM, Abraham A, Bianco S, Esposito A (2022). Loop-extrusion and polymer phase-separation can co-exist at the single-molecule level to shape chromatin folding. Nat Commun.

[CR42] Cowan CR, Carlton PM, Cande WZ (2001). The polar arrangement of telomeres in interphase and meiosis. Rabl organization and the bouquet. Plant Physiol.

[CR43] Criscione SW, De Cecco M, Siranosian B, Zhang Y, Kreiling JA, Sedivy JM (2016). Reorganization of chromosome architecture in replicative cellular senescence. Sci Adv.

[CR44] Crump NT, Ballabio E, Godfrey L, Thorne R, Repapi E, Kerry J (2021). BET inhibition disrupts transcription but retains enhancer-promoter contact. Nat Commun.

[CR45] Cruz-Molina S, Respuela P, Tebartz C, Kolovos P, Nikolic M, Fueyo R (2017). PRC2 facilitates the regulatory topology required for poised enhancer function during pluripotent stem cell differentiation. Cell Stem Cell.

[CR46] Dall’Agnese A, Caputo L, Nicoletti C, di Iulio J, Schmitt A, Gatto S (2019). Transcription factor-directed re-wiring of chromatin architecture for somatic cell nuclear reprogramming toward trans-differentiation. Mol Cell.

[CR47] Daneshvar K, Ardehali MB, Klein IA, Hsieh FK, Kratkiewicz AJ, Mahpour A (2020). lncRNA DIGIT and BRD3 protein form phase-separated condensates to regulate endoderm differentiation. Nat Cell Biol.

[CR48] Davidson IF, Peters JM (2021). Genome folding through loop extrusion by SMC complexes. Nat Rev Mol Cell Biol.

[CR49] Dekker J, Rippe K, Dekker M, Kleckner N (2002). Capturing chromosome conformation. Science.

[CR50] Di Giammartino DC, Kloetgen A, Polyzos A, Liu Y, Kim D, Murphy D (2019). KLF4 is involved in the organization and regulation of pluripotency-associated three-dimensional enhancer networks. Nat Cell Biol.

[CR51] Di Pierro M, Zhang B, Aiden EL, Wolynes PG, Onuchic JN (2016). Transferable model for chromosome architecture. Proc Natl Acad Sci U S A.

[CR52] Di Stefano M, Stadhouders R, Farabella I, Castillo D, Serra F, Graf T (2020). Transcriptional activation during cell reprogramming correlates with the formation of 3D open chromatin hubs. Nat Commun.

[CR53] Dixon JR, Jung I, Selvaraj S, Shen Y, Antosiewicz-Bourget JE, Lee AY (2015). Chromatin architecture reorganization during stem cell differentiation. Nature.

[CR54] Dixon JR, Selvaraj S, Yue F, Kim A, Li Y, Shen Y (2012). Topological domains in mammalian genomes identified by analysis of chromatin interactions. Nature.

[CR55] Dixon JR, Xu J, Dileep V, Zhan Y, Song F, Le VT (2018). Integrative detection and analysis of structural variation in cancer genomes. Nat Genet.

[CR56] Dodson AE, Kennedy S (2020). Phase separation in germ cells and development. Dev Cell.

[CR57] Dostie J, Richmond TA, Arnaout RA, Selzer RR, Lee WL, Honan TA (2006). Chromosome conformation capture carbon copy (5C): a massively parallel solution for mapping interactions between genomic elements. Genome Res.

[CR58] Du Z, Zheng H, Huang B, Ma R, Wu J, Zhang X (2017). Allelic reprogramming of 3D chromatin architecture during early mammalian development. Nature.

[CR59] Du Z, Zheng H, Kawamura YK, Zhang K, Gassler J, Powell S (2020). Polycomb group proteins regulate chromatin architecture in mouse oocytes and early embryos. Mol Cell.

[CR60] Duan Z, Andronescu M, Schutz K, McIlwain S, Kim YJ, Lee C (2010). A three-dimensional model of the yeast genome. Nature.

[CR61] El Khattabi L, Zhao H, Kalchschmidt J, Young N, Jung S, Van Blerkom P (2019). A pliable mediator acts as a functional rather than an architectural bridge between promoters and enhancers. Cell.

[CR62] Elbaum-Garfinkle S (2019). Matter over mind: liquid phase separation and neurodegeneration. J Biol Chem.

[CR63] Erdel F, Rademacher A, Vlijm R, Tunnermann J, Frank L, Weinmann R (2020). Mouse heterochromatin adopts digital compaction states without showing hallmarks of HP1-driven liquid-liquid phase separation. Mol Cell.

[CR64] Erdel F, Rippe K (2018). Formation of chromatin subcompartments by phase separation. Biophys J.

[CR65] Esposito A, Bianco S, Chiariello AM, Abraham A, Fiorillo L, Conte M (2022). Polymer physics reveals a combinatorial code linking 3D chromatin architecture to 1D chromatin states. Cell Rep.

[CR66] Falahati H, Pelham-Webb B, Blythe S, Wieschaus E (2016). Nucleation by rRNA dictates the precision of nucleolus assembly. Curr Biol.

[CR67] Fasciani A, D’Annunzio S, Poli V, Fagnocchi L, Beyes S, Michelatti D (2020). MLL4-associated condensates counterbalance Polycomb-mediated nuclear mechanical stress in Kabuki syndrome. Nat Genet.

[CR68] Flavahan WA, Drier Y, Liau BB, Gillespie SM, Venteicher AS, Stemmer-Rachamimov AO (2016). Insulator dysfunction and oncogene activation in IDH mutant gliomas. Nature.

[CR69] Flyamer IM, Gassler J, Imakaev M, Brandao HB, Ulianov SV, Abdennur N (2017). Single-nucleus Hi-C reveals unique chromatin reorganization at oocyte-to-zygote transition. Nature.

[CR70] Franke M, Ibrahim DM, Andrey G, Schwarzer W, Heinrich V, Schöpflin R (2016). Formation of new chromatin domains determines pathogenicity of genomic duplications. Nature.

[CR71] Fraser J, Ferrai C, Chiariello AM, Schueler M, Rito T, Laudanno G (2015). Hierarchical folding and reorganization of chromosomes are linked to transcriptional changes in cellular differentiation. Mol Syst Biol.

[CR72] Fudenberg G, Imakaev M, Lu C, Goloborodko A, Abdennur N, Mirny Leonid A (2016). Formation of chromosomal domains by loop extrusion. Cell Rep.

[CR73] Gibson BA, Doolittle LK, Schneider MWG, Jensen LE, Gamarra N, Henry L (2019). Organization of chromatin by intrinsic and regulated phase separation. Cell.

[CR74] Graf T, Enver T (2009). Forcing cells to change lineages. Nature.

[CR75] Grosch M, Ittermann S, Shaposhnikov D, Drukker M (2020). Chromatin-associated membraneless organelles in regulation of cellular differentiation. Stem Cell Rep.

[CR76] Groschel S, Sanders MA, Hoogenboezem R, de Wit E, Bouwman BAM, Erpelinck C (2014). A single oncogenic enhancer rearrangement causes concomitant EVI1 and GATA2 deregulation in leukemia. Cell.

[CR77] Heyn P, Salmonowicz H, Rodenfels J, Neugebauer KM (2017). Activation of transcription enforces the formation of distinct nuclear bodies in zebrafish embryos. RNA Biol.

[CR78] Hnisz D, Weintraub AS, Day DS, Valton AL, Bak RO, Li CH (2016). Activation of proto-oncogenes by disruption of chromosome neighborhoods. Science.

[CR79] Hu S, Lv P, Yan Z, Wen B (2019). Disruption of nuclear speckles reduces chromatin interactions in active compartments. Epigenetics Chromatin.

[CR80] Hug CB, Grimaldi AG, Kruse K, Vaquerizas JM (2017). Chromatin architecture emerges during zygotic genome activation independent of transcription. Cell.

[CR81] Hug CB, Vaquerizas JM (2018). The birth of the 3D genome during early embryonic development. Trends Genet.

[CR82] Jachowicz JW, Strehle M, Banerjee AK, Blanco MR, Thai J, Guttman M (2022). Xist spatially amplifies SHARP/SPEN recruitment to balance chromosome-wide silencing and specificity to the X chromosome. Nat Struct Mol Biol.

[CR83] Jerkovic I, Cavalli G (2021). Understanding 3D genome organization by multidisciplinary methods. Nat Rev Mol Cell Biol.

[CR84] Jones PL, Veenstra GJ, Wade PA, Vermaak D, Kass SU, Landsberger N (1998). Methylated DNA and MeCP2 recruit histone deacetylase to repress transcription. Nat Genet.

[CR85] Joshi O, Wang SY, Kuznetsova T, Atlasi Y, Peng T, Fabre PJ (2015). Dynamic reorganization of extremely long-range promoter-promoter interactions between two states of pluripotency. Cell Stem Cell.

[CR86] Jucker M, Walker LC (2013). Self-propagation of pathogenic protein aggregates in neurodegenerative diseases. Nature..

[CR87] Jukam D, Shariati SAM, Skotheim JM (2017). Zygotic genome activation in vertebrates. Dev Cell.

[CR88] Kaaij LJT, van der Weide RH, Ketting RF, de Wit E (2018). Systemic loss and gain of chromatin architecture throughout zebrafish development. Cell Rep.

[CR89] Ke Y, Xu Y, Chen X, Feng S, Liu Z, Sun Y (2017). 3D chromatin structures of mature gametes and structural reprogramming during mammalian embryogenesis. Cell.

[CR90] Kempfer R, Pombo A (2020). Methods for mapping 3D chromosome architecture. Nat Rev Genet.

[CR91] Kragesteen BK, Spielmann M, Paliou C, Heinrich V, Schopflin R, Esposito A (2018). Dynamic 3D chromatin architecture contributes to enhancer specificity and limb morphogenesis. Nat Genet.

[CR92] Kroschwald S, Maharana S, Simon A. Hexanediol: a chemical probe to investigate the material properties of membrane-less compartments. Matters. 2017. 10.19185/matters.201702000010.

[CR93] Kuang J, Zhai Z, Li P, Shi R, Guo W, Yao Y (2021). SS18 regulates pluripotent-somatic transition through phase separation. Nat Commun.

[CR94] Lafontaine DLJ, Riback JA, Bascetin R, Brangwynne CP (2021). The nucleolus as a multiphase liquid condensate. Nat Rev Mol Cell Biol.

[CR95] Larson AG, Elnatan D, Keenen MM, Trnka MJ, Johnston JB, Burlingame AL (2017). Liquid droplet formation by HP1alpha suggests a role for phase separation in heterochromatin. Nature.

[CR96] Larson JD, Kasper LH, Paugh BS, Jin H, Wu G, Kwon CH (2019). Histone H3.3 K27M accelerates spontaneous brainstem glioma and drives restricted changes in bivalent gene expression. Cancer Cell.

[CR97] Lee DP, Tan WLW, Anene-Nzelu CG, Lee CJM, Li PY, Luu TDA (2019). Robust CTCF-based chromatin architecture underpins epigenetic changes in the heart failure stress-gene response. Circulation.

[CR98] Lee J, Termglinchan V, Diecke S, Itzhaki I, Lam CK, Garg P (2019). Activation of PDGF pathway links LMNA mutation to dilated cardiomyopathy. Nature.

[CR99] Lettice LA, Heaney SJ, Purdie LA, Li L, de Beer P, Oostra BA (2003). A long-range Shh enhancer regulates expression in the developing limb and fin and is associated with preaxial polydactyly. Hum Mol Genet.

[CR100] Li F, An Z, Zhang Z. The dynamic 3D genome in gametogenesis and early embryonic development. Cells. 2019;8(8). 10.3390/cells8080788. 10.3390/cells8080788 PMC6721571 31362461

[CR101] Li R, Liu Y, Hou Y, Gan J, Wu P, Li C (2018). 3D genome and its disorganization in diseases. Cell Biol Toxicol.

[CR102] Lieberman-Aiden E, van Berkum NL, Williams L, Imakaev M, Ragoczy T, Telling A (2009). Comprehensive mapping of long-range interactions reveals folding principles of the human genome. Science.

[CR103] Linares-Saldana R, Kim W, Bolar NA, Zhang H, Koch-Bojalad BA, Yoon S (2021). BRD4 orchestrates genome folding to promote neural crest differentiation. Nat Genet.

[CR104] Liu J, Wang L, Wang Z, Liu JP. Roles of telomere biology in cell senescence, replicative and chronological ageing. Cells. 2019;8(1). 10.3390/cells8010054. 10.3390/cells8010054 PMC6356700 30650660

[CR105] Liu X, Jiang S, Ma L, Qu J, Zhao L, Zhu X (2021). Time-dependent effect of 1,6-hexanediol on biomolecular condensates and 3D chromatin organization. Genome Biol.

[CR106] Liu X, Liu X, Wang H, Dou Z, Ruan K, Hill DL (2020). Phase separation drives decision making in cell division. J Biol Chem.

[CR107] Liu X, Shen J, Xie L, Wei Z, Wong C, Li Y (2020). Mitotic implantation of the transcription factor prospero via phase separation drives terminal neuronal differentiation. Dev Cell.

[CR108] Lu C, Jain SU, Hoelper D, Bechet D, Molden RC, Ran L (2016). Histone H3K36 mutations promote sarcomagenesis through altered histone methylation landscape. Science.

[CR109] Ma X, Cao X, Zhu L, Li Y, Wang X, Wu B, et al. Pre-existing chromatin accessibility of switchable repressive compartment delineates cell plasticity. Natl Sci Rev. 2021. 10.1093/nsr/nwab230. 10.1093/nsr/nwab230 PMC9249582 35795460

[CR110] Machyna M, Heyn P, Neugebauer KM (2013). Cajal bodies: where form meets function. Wiley Interdiscip Rev RNA.

[CR111] Mansour MR, Abraham BJ, Anders L, Berezovskaya A, Gutierrez A, Durbin AD (2014). Oncogene regulation. An oncogenic super-enhancer formed through somatic mutation of a noncoding intergenic element. Science.

[CR112] Marlow FL, Mullins MC (2008). Bucky ball functions in Balbiani body assembly and animal-vegetal polarity in the oocyte and follicle cell layer in zebrafish. Dev Biol.

[CR113] Martin P, McGovern A, Orozco G, Duffus K, Yarwood A, Schoenfelder S (2015). Capture Hi-C reveals novel candidate genes and complex long-range interactions with related autoimmune risk loci. Nat Commun.

[CR114] McHugh CA, Chen CK, Chow A, Surka CF, Tran C, McDonel P (2015). The Xist lncRNA interacts directly with SHARP to silence transcription through HDAC3. Nature.

[CR115] McSwiggen DT, Hansen AS, Teves SS, Marie-Nelly H, Hao Y, Heckert AB, et al. Evidence for DNA-mediated nuclear compartmentalization distinct from phase separation. Elife. 2019;8. 10.7554/eLife.47098. 10.7554/eLife.47098 PMC6522219 31038454

[CR116] Monahan K, Horta A, Lomvardas S (2019). LHX2- and LDB1-mediated trans interactions regulate olfactory receptor choice. Nature.

[CR117] Morgan MAJ, Shilatifard A (2020). Reevaluating the roles of histone-modifying enzymes and their associated chromatin modifications in transcriptional regulation. Nat Genet.

[CR118] Maslova A, Krasikova A (2021). FISH Going Meso-Scale: A Microscopic Search for Chromatin Domains..

[CR119] Nagulapalli M, Maji S, Dwivedi N, Dahiya P, Thakur JK (2016). Evolution of disorder in mediator complex and its functional relevance. Nucleic Acids Res.

[CR120] Nakamura R, Motai Y, Kumagai M, Wike CL, Nishiyama H, Nakatani Y (2021). CTCF looping is established during gastrulation in medaka embryos. Genome Res.

[CR121] Nan X, Ng HH, Johnson CA, Laherty CD, Turner BM, Eisenman RN (1998). Transcriptional repression by the methyl-CpG-binding protein MeCP2 involves a histone deacetylase complex. Nature.

[CR122] Narita M, Nũnez S, Heard E, Narita M, Lin AW, Hearn SA (2003). Rb-mediated heterochromatin formation and silencing of E2F target genes during cellular senescence. Cell.

[CR123] Nicodemi M, Prisco A (2009). Thermodynamic pathways to genome spatial organization in the cell nucleus. Biophys J.

[CR124] Niu L, Shen W, Shi Z, Tan Y, He N, Wan J (2021). Three-dimensional folding dynamics of the Xenopus tropicalis genome. Nat Genet.

[CR125] Nora EP, Lajoie BR, Schulz EG, Giorgetti L, Okamoto I, Servant N (2012). Spatial partitioning of the regulatory landscape of the X-inactivation centre. Nature.

[CR126] Northcott PA, Lee C, Zichner T, Stütz AM, Erkek S, Kawauchi D (2014). Enhancer hijacking activates GFI1 family oncogenes in medulloblastoma. Nature.

[CR127] Paliou C, Guckelberger P, Schöpflin R, Heinrich V, Esposito A, Chiariello AM (2019). Preformed chromatin topology assists transcriptional robustness of Shh during limb development. Proc Natl Acad Sci U S A.

[CR128] Pandya-Jones A, Markaki Y, Serizay J, Chitiashvili T, Mancia Leon WR, Damianov A (2020). A protein assembly mediates Xist localization and gene silencing. Nature.

[CR129] Peskett TR, Rau F, O’Driscoll J, Patani R, Lowe AR, Saibil HR (2018). A liquid to solid phase transition underlying pathological huntingtin exon1 aggregation. Mol Cell.

[CR130] Polymeropoulos MH, Lavedan C, Leroy E, Ide SE, Dehejia A, Dutra A (1997). Mutation in the alpha-synuclein gene identified in families with Parkinson’s disease. Science.

[CR131] Protter DSW, Parker R (2016). Principles and properties of stress granules. Trends Cell Biol.

[CR132] Quinodoz SA, Jachowicz JW, Bhat P, Ollikainen N, Banerjee AK, Goronzy IN (2021). RNA promotes the formation of spatial compartments in the nucleus. Cell.

[CR133] Quinodoz SA, Ollikainen N, Tabak B, Palla A, Schmidt JM, Detmar E (2018). Higher-order inter-chromosomal hubs shape 3D genome organization in the nucleus. Cell.

[CR134] Quiroz FG, Fiore VF, Levorse J, Polak L, Wong E, Pasolli HA, et al. Liquid-liquid phase separation drives skin barrier formation. Science. 2020;367(6483). 10.1126/science.aax9554. 10.1126/science.aax9554 PMC7258523 32165560

[CR135] Rao SS, Huntley MH, Durand NC, Stamenova EK, Bochkov ID, Robinson JT (2014). A 3D map of the human genome at kilobase resolution reveals principles of chromatin looping. Cell.

[CR136] Reik W (2007). Stability and flexibility of epigenetic gene regulation in mammalian development. Nature.

[CR137] Roden C, Gladfelter AS (2021). RNA contributions to the form and function of biomolecular condensates. Nat Rev Mol Cell Biol.

[CR138] Rosa-Garrido M, Chapski DJ, Schmitt AD, Kimball TH, Karbassi E, Monte E (2017). High-resolution mapping of chromatin conformation in cardiac myocytes reveals structural remodeling of the epigenome in heart failure. Circulation.

[CR139] Ryu JK, Bouchoux C, Liu HW, Kim E, Minamino M, de Groot R, et al. Bridging-induced phase separation induced by cohesin SMC protein complexes. Sci Adv. 2021;7(7). 10.1126/sciadv.abe5905. 10.1126/sciadv.abe5905 PMC7875533 33568486

[CR140] Sabari BR, Dall’Agnese A, Boija A, Klein IA, Coffey EL, Shrinivas K, et al. Coactivator condensation at super-enhancers links phase separation and gene control. Science. 2018;361(6400). 10.1126/science.aar3958. 10.1126/science.aar3958 PMC6092193 29930091

[CR141] Sabari BR, Dall’Agnese A, Young RA (2020). Biomolecular condensates in the nucleus. Trends Biochem Sci.

[CR142] Sanulli S, Trnka MJ, Dharmarajan V, Tibble RW, Pascal BD, Burlingame AL (2019). HP1 reshapes nucleosome core to promote phase separation of heterochromatin. Nature.

[CR143] Sati S, Bonev B, Szabo Q, Jost D, Bensadoun P, Serra F (2020). 4D genome rewiring during oncogene-induced and replicative senescence. Mol Cell.

[CR144] Sawyer IA, Shevtsov SP, Dundr M (2016). Spectral imaging to visualize higher-order genomic organization. Nucleus.

[CR145] Sawyer IA, Sturgill D, Sung MH, Hager GL, Dundr M (2016). Cajal body function in genome organization and transcriptome diversity. Bioessays.

[CR146] Schmitt AD, Hu M, Jung I, Xu Z, Qiu Y, Tan CL (2016). A compendium of chromatin contact maps reveals spatially active regions in the human genome. Cell Rep.

[CR147] Schoenfelder S, Sugar R, Dimond A, Javierre BM, Armstrong H, Mifsud B (2015). Polycomb repressive complex PRC1 spatially constrains the mouse embryonic stem cell genome. Nat Genet.

[CR148] Schumacher B, Pothof J, Vijg J, Hoeijmakers JHJ (2021). The central role of DNA damage in the ageing process. Nature.

[CR149] Sexton T, Yaffe E, Kenigsberg E, Bantignies F, Leblanc B, Hoichman M (2012). Three-dimensional folding and functional organization principles of the Drosophila genome. Cell.

[CR150] Sharma R, Choi KJ, Quan MD, Sharma S, Sankaran B, Park H (2021). Liquid condensation of reprogramming factor KLF4 with DNA provides a mechanism for chromatin organization. Nat Commun.

[CR151] Shi B, Li W, Song Y, Wang Z, Ju R, Ulman A (2021). UTX condensation underlies its tumour-suppressive activity. Nature.

[CR152] Shin JY, Worman HJ (2022). Molecular pathology of laminopathies. Annu Rev Pathol.

[CR153] Shin Y, Chang YC, Lee DSW, Berry J, Sanders DW, Ronceray P (2019). Liquid nuclear condensates mechanically sense and restructure the genome. Cell.

[CR154] Shoji M, Tanaka T, Hosokawa M, Reuter M, Stark A, Kato Y (2009). The TDRD9-MIWI2 complex is essential for piRNA-mediated retrotransposon silencing in the mouse male germline. Dev Cell.

[CR155] Siersbaek R, Madsen JGS, Javierre BM, Nielsen R, Bagge EK, Cairns J (2017). Dynamic rewiring of promoter-anchored chromatin loops during adipocyte differentiation. Mol Cell.

[CR156] Simonis M, Klous P, Splinter E, Moshkin Y, Willemsen R, de Wit E (2006). Nuclear organization of active and inactive chromatin domains uncovered by chromosome conformation capture-on-chip (4C). Nat Genet.

[CR157] So C, Cheng S, Schuh M (2021). Phase separation during germline development. Trends Cell Biol.

[CR158] Spannl S, Tereshchenko M, Mastromarco GJ, Ihn SJ, Lee HO (2019). Biomolecular condensates in neurodegeneration and cancer. Traffic.

[CR159] Spielmann M, Lupianez DG, Mundlos S (2018). Structural variation in the 3D genome. Nat Rev Genet.

[CR160] Spillantini MG, Schmidt ML, Lee VM, Trojanowski JQ, Jakes R, Goedert M (1997). Alpha-synuclein in Lewy bodies. Nature.

[CR161] Stadhouders R, Vidal E, Serra F, Di Stefano B, Le Dily F, Quilez J (2018). Transcription factors orchestrate dynamic interplay between genome topology and gene regulation during cell reprogramming. Nat Genet.

[CR162] Stevens TJ, Lando D, Basu S, Atkinson LP, Cao Y, Lee SF (2017). 3D structures of individual mammalian genomes studied by single-cell Hi-C. Nature.

[CR163] Stik G, Vidal E, Barrero M, Cuartero S, Vila-Casadesus M, Mendieta-Esteban J (2020). CTCF is dispensable for immune cell transdifferentiation but facilitates an acute inflammatory response. Nat Genet.

[CR164] Strom AR, Emelyanov AV, Mir M, Fyodorov DV, Darzacq X, Karpen GH (2017). Phase separation drives heterochromatin domain formation. Nature.

[CR165] Strzelecka M, Oates AC, Neugebauer KM (2010). Dynamic control of Cajal body number during zebrafish embryogenesis. Nucleus.

[CR166] Su JH, Zheng P, Kinrot SS, Bintu B, Zhuang X (2020). Genome-scale imaging of the 3D organization and transcriptional activity of chromatin. Cell.

[CR167] Sun Q, Perez-Rathke A, Czajkowsky DM, Shao Z, Liang J (2021). High-resolution single-cell 3D-models of chromatin ensembles during Drosophila embryogenesis. Nat Commun.

[CR168] Symmons O, Uslu VV, Tsujimura T, Ruf S, Nassari S, Schwarzer W (2014). Functional and topological characteristics of mammalian regulatory domains. Genome Res.

[CR169] Taberlay PC, Achinger-Kawecka J, Lun AT, Buske FA, Sabir K, Gould CM (2016). Three-dimensional disorganization of the cancer genome occurs coincident with long-range genetic and epigenetic alterations. Genome Res.

[CR170] Takahashi K, Yamanaka S (2006). Induction of pluripotent stem cells from mouse embryonic and adult fibroblast cultures by defined factors. Cell.

[CR171] Tatavosian R, Kent S, Brown K, Yao T, Duc HN, Huynh TN (2019). Nuclear condensates of the Polycomb protein chromobox 2 (CBX2) assemble through phase separation. J Biol Chem.

[CR172] Tatomer DC, Terzo E, Curry KP, Salzler H, Sabath I, Zapotoczny G (2016). Concentrating pre-mRNA processing factors in the histone locus body facilitates efficient histone mRNA biogenesis. J Cell Biol.

[CR173] Trcek T, Lehmann R (2019). Germ granules in Drosophila. Traffic.

[CR174] Trojanowski J, Frank L, Rademacher A, Mucke N, Grigaitis P, Rippe K (2022). Transcription activation is enhanced by multivalent interactions independent of phase separation. Mol Cell.

[CR175] Vallot A, Tachibana K (2020). The emergence of genome architecture and zygotic genome activation. Curr Opin Cell Biol.

[CR176] Vara C, Paytuvi-Gallart A, Cuartero Y, Le Dily F, Garcia F, Salva-Castro J (2019). Three-dimensional genomic structure and cohesin occupancy correlate with transcriptional activity during spermatogenesis. Cell Rep.

[CR177] Wang J, Yu H, Ma Q, Zeng P, Wu D, Hou Y (2021). Phase separation of OCT4 controls TAD reorganization to promote cell fate transitions. Cell Stem Cell.

[CR178] Wang L, Gao Y, Zheng X, Liu C, Dong S, Li R (2019). Histone modifications regulate chromatin compartmentalization by contributing to a phase separation mechanism. Mol Cell.

[CR179] Wang L, Hu M, Zuo MQ, Zhao J, Wu D, Huang L (2020). Rett syndrome-causing mutations compromise MeCP2-mediated liquid-liquid phase separation of chromatin. Cell Res.

[CR180] Wang Q, Sawyer IA, Sung MH, Sturgill D, Shevtsov SP, Pegoraro G (2016). Cajal bodies are linked to genome conformation. Nat Commun.

[CR181] Wang S, Su JH, Beliveau BJ, Bintu B, Moffitt JR, Wu CT (2016). Spatial organization of chromatin domains and compartments in single chromosomes. Science.

[CR182] Wang W, Qiao S, Li G, Cheng J, Yang C, Zhong C, et al. A histidine cluster determines YY1-compartmentalized coactivators and chromatin elements in phase-separated enhancer clusters. Nucleic Acids Res. 2022. 10.1093/nar/gkac233. 10.1093/nar/gkac233 PMC9122595 35390165

[CR183] Wang Y, Wang H, Zhang Y, Du Z, Si W, Fan S (2019). Reprogramming of meiotic chromatin architecture during spermatogenesis. Mol Cell.

[CR184] Wei C, Jia L, Huang X, Tan J, Wang M, Niu J, et al. CTCF organizes inter-A compartment interactions through RYBP-dependent phase separation. Cell Res. 2022. 10.1038/s41422-022-00676-0. 10.1038/s41422-022-00676-0 PMC9343660 35768498

[CR185] Weischenfeldt J, Dubash T, Drainas AP, Mardin BR, Chen Y, Stutz AM (2017). Pan-cancer analysis of somatic copy-number alterations implicates IRS4 and IGF2 in enhancer hijacking. Nat Genet.

[CR186] Whyte WA, Orlando DA, Hnisz D, Abraham BJ, Lin CY, Kagey MH (2013). Master transcription factors and mediator establish super-enhancers at key cell identity genes. Cell.

[CR187] Wike CL, Guo Y, Tan M, Nakamura R, Shaw DK, Diaz N (2021). Chromatin architecture transitions from zebrafish sperm through early embryogenesis. Genome Res.

[CR188] Williamson I, Lettice LA, Hill RE, Bickmore WA (2016). Shh and ZRS enhancer colocalisation is specific to the zone of polarising activity. Development.

[CR189] Wu P, Li T, Li R, Jia L, Zhu P, Liu Y (2017). 3D genome of multiple myeloma reveals spatial genome disorganization associated with copy number variations. Nat Commun.

[CR190] Zatsepina O, Baly C, Chebrout M, Debey P (2003). The step-wise assembly of a functional nucleolus in preimplantation mouse embryos involves the cajal (coiled) body. Dev Biol.

[CR191] Zenk F, Zhan Y, Kos P, Löser E, Atinbayeva N, Schächtle M (2021). HP1 drives de novo 3D genome reorganization in early Drosophila embryos. Nature.

[CR192] Zhang C, Xu Z, Yang S, Sun G, Jia L, Zheng Z (2020). tagHi-C reveals 3D chromatin architecture dynamics during mouse hematopoiesis. Cell Rep.

[CR193] Zhang Y, Li T, Preissl S, Amaral ML, Grinstein JD, Farah EN (2019). Transcriptionally active HERV-H retrotransposons demarcate topologically associating domains in human pluripotent stem cells. Nat Genet.

[CR194] Zhao K, Wang M, Gao S, Chen J (2021). Chromatin architecture reorganization during somatic cell reprogramming. Curr Opin Genet Dev.

[CR195] Zhao Z, Tavoosidana G, Sjölinder M, Göndör A, Mariano P, Wang S (2006). Circular chromosome conformation capture (4C) uncovers extensive networks of epigenetically regulated intra- and interchromosomal interactions. Nat Genet.

[CR196] Zheng H, Xie W (2019). The role of 3D genome organization in development and cell differentiation. Nat Rev Mol Cell Biol.

